# Multiple Score Comparison: a network meta-analysis approach to comparison and external validation of prognostic scores

**DOI:** 10.1186/s12874-017-0433-2

**Published:** 2017-12-21

**Authors:** Sarah R. Haile, Beniamino Guerra, Joan B. Soriano, Milo A. Puhan, for the 3CIA collaboration, Bernd Lamprecht, Ana S. Ramírez, Pablo Martinez-Camblor, Bernhard Kaiser, Inmaculada Alfageme, Pere Almagro, Ciro Casanova, Cristóbal Esteban-González, Juan J. Soler-Cataluña, Juan P. de Torres, Marc. Miravitlles, Bartolome R. Celli, Jose M. Marin, Gerben ter Riet, Patricia Sobradillo-Ecenarro, Peter Lange, Judith Garcia-Aymerich, Josep M. Antó-Boqué, Alice M. Turner, Meilan K. Han, Arnulf Langhammer, Linda Leivseth, Per Bakke, Ane Johannessen, Oga Toru, Borja Cosío, Julio Ancochea-Bermúdez, Andres Echazarreta, Nicolas Roche, Pierre-Régis Burgel, Don D. Sin, Joan B. Soriano, Milo A. Puhan

**Affiliations:** 1 Epidemiology, Biostatistics and Prevention Institute, University of Zurich, Zurich, Switzerland; 2 Servicio de Neumología, Instituto de Investigación del Hospital Universitario de la Princesa (IISP), Universidad Autónoma de Madrid, Madrid, Spain; 3 Epidemiology & Department of Epidemiology, Johns Hopkins Bloomberg School of Public Health, Baltimore, USA

**Keywords:** Prognostic scores, External validation, Multiple score comparison, Chronic obstructive pulmonary disease

## Abstract

**Background:**

Prediction models and prognostic scores have been increasingly popular in both clinical practice and clinical research settings, for example to aid in risk-based decision making or control for confounding. In many medical fields, a large number of prognostic scores are available, but practitioners may find it difficult to choose between them due to lack of external validation as well as lack of comparisons between them.

**Methods:**

Borrowing methodology from network meta-analysis, we describe an approach to Multiple Score Comparison meta-analysis (MSC) which permits concurrent external validation and comparisons of prognostic scores using individual patient data (IPD) arising from a large-scale international collaboration. We describe the challenges in adapting network meta-analysis to the MSC setting, for instance the need to explicitly include correlations between the scores on a cohort level, and how to deal with many multi-score studies. We propose first using IPD to make cohort-level aggregate discrimination or calibration scores, comparing all to a common comparator. Then, standard network meta-analysis techniques can be applied, taking care to consider correlation structures in cohorts with multiple scores. Transitivity, consistency and heterogeneity are also examined.

**Results:**

We provide a clinical application, comparing prognostic scores for 3-year mortality in patients with chronic obstructive pulmonary disease using data from a large-scale collaborative initiative. We focus on the discriminative properties of the prognostic scores. Our results show clear differences in performance, with ADO and eBODE showing higher discrimination with respect to mortality than other considered scores. The assumptions of transitivity and local and global consistency were not violated. Heterogeneity was small.

**Conclusions:**

We applied a network meta-analytic methodology to externally validate and concurrently compare the prognostic properties of clinical scores. Our large-scale external validation indicates that the scores with the best discriminative properties to predict 3 year mortality in patients with COPD are ADO and eBODE.

**Electronic supplementary material:**

The online version of this article (doi:10.1186/s12874-017-0433-2) contains supplementary material, which is available to authorized users.

## Background

Prediction models, which combine predictors using regression coefficients, and simpler prognostic scores, which typically assign point values to predictors based on prediction models, have become increasingly popular [1, 2]. They aid in decision making in public health, clinical research and clinical practice [3] by estimating a person’s risk of developing a disease or other outcome. In several medical fields, a variety of prediction models have been developed to assess the individual risk of adverse outcomes. A great example for this was a very recent systematic review regarding validated risk factor models for neurodevelopmental outcomes in children born very preterm or with very low birth weight [4]; 78 original studies (including 222 prediction models) were extracted. Most of the models were not intended to be used for clinical practice and only four studies (5%) had performed a validation. Another example regards models predicting risk of type 2 diabetes mellitus with genetic risk models on the basis of established genome-wide association markers; a systematic review deemed to be eligible 21 articles representing 23 studies [5]. Concerning the risk of developing cardiovascular disease, over the past two decades, numerous prediction models have been developed, to estimate the risk of developing cardiovascular disease [6]. Only 36% of them were externally validated and only 19% by independent investigators. In the case of chronic obstructive pulmonary disease (COPD), several prognostic scores have been developed to predict mortality, starting with the BODE score [7]. But scores also exist to predict exacerbations [8], or the course of health-related quality of life [9, 10]. Prognostic scores suffer from a reluctance of general practitioners to use them [11, 12] as well as from scepticism because they lack internal and external validation which are requirements for generalizability [13, 14]. The external validation studies are often simply poorly designed or reported [15]. The lack of comparisons among available prognostic scores provides an additional hurdle to their widespread applicability, as practitioners may not be able to decide among them based on the information available [16].

Luckily, the collection of “big data” [17] and the growing availability of individual patient data (IPD) data analyses [18–23] provide researchers with new opportunities and challenges [24, 25]. Furthermore, the call of the medical community for data sharing [26] improves the possibilities of checking a model’s predictive performance across clinical settings, populations, and subgroups [25]. The COCOMICS study [27] is a rare example of prognostic scores being directly compared with each other and simultaneously externally validated after pooling all the databases in a single cohort [16]. Our approach, multiple score comparison network meta-analysis (MSC), extends the simple pooling approach to pool direct comparisons taken from different studies, as a meta-analysis across studies provides in general higher quality information compared to the analysis of a database, constituted pooling together the single studies [28, 29]. This methodology allows to take into account heterogeneity of the individual studies and obtain more generalizable results [25].

## Methods

Various methodological approaches have been proposed for network meta-analysis for comparison of treatments [30–36], which is sometimes referred to as network meta-analysis, multiple (or mixed) treatment comparisons meta-analysis (MTC meta-analysis) or multiple treatments meta-analysis [37, 38]. For diagnostic test performance, the first steps of network meta-analysis were undertaken (e.g. in terms of sensitivity and specificity) [39, 40]. No similar methodology exists to compare the performance of prognostic scores or prediction models. Nevertheless, network meta-analysis may provide an attractive solution to the problem of comparing the performance of prognostic scores.

Changing from comparing effects of treatments to comparing performance of prediction models or prognostic scores, however, reveals a number of differences between the two settings, and care must be taken to ensure that the unique features of multiple score comparison (MSC) meta-analysis (as we will refer to this new method) are considered properly in the analysis.

A number of features distinguish a MSC meta-analysis of prognostic scores from a meta-analysis of treatments. In network meta-analysis of treatments outcomes are summarized separately within each treatment arm of a randomized trial, and combined to obtain estimates of treatment effect (for example, mean difference or log odds ratio); instead, the MSC meta-analysis uses performance measures of each score in a cohort that can be calculated on the same sample of patients. Additionally, the number of prognostic scores assessed in a given cohort is not limited by the practicalities of study design, so that it would be easily possible to have more than, say, four scores within one cohort, while such a large number of treatment arms in an RCT is relatively unlikely due to considerations of power and sample size along with practical aspects of conducting clinical trials. Consideration of multi-score studies properly, including the correlations inherent in such comparisons, in MSC is therefore of great importance.

We developed a comprehensive approach to MSC to assess various prediction models using network meta-analysis with individual patient data, providing external validation and concurrent comparison of the scores, and applied it to risk prediction scores for mortality in COPD [41, 42]. After careful methodological issues (see also online-only material, where we go deeper into the statistical background) the following approach was developed: we calculated aggregated summary statistics for each cohort and score. Then, we examined the network structure by grouping the cohorts according to which scores could be evaluated. We adapted methodology from network meta-analysis [35] to concurrently externally validate and compare prognostic scores from individual patient data across different cohorts, explicitly including correlations [43] between the scores on a cohort level.

We will also re-interpret NMA as a two-stage meta-regression model, as proposed in [35]:

1. Ordinary meta-analysis to gain the direct estimates for corresponding pooled effect estimates (using the inverse-variance weighted means of the corresponding cohorts). Cohorts at our disposal are classified into “groups” according to which scores it is possible to evaluate by their data.
2. Based on the direct estimates and their variances from the first stage, they obtain to find the optimal estimate of the pooled effect parameters that obeys the fundamental consistency equations. In this stage we merge the group estimates, looking for the weighted least squares solution to the regression problem equation.

The last steps were to confirm that transitivity is a plausible assumption and to check for possible inconsistency and heterogeneity.

### Calculation of aggregated summary statistics

First, the performance measure for comparison of the various prognostic scores was defined as the area under the curve (AUC) of the corresponding receiver operating characteristic curve (ROC). This is a graphical plot that illustrates the ability of a binary classifier system (diagnostic or prognostic) as its discrimination threshold is varied (in particular plotting true vs false positive rate). Differences in AUC, denoted ΔAUC, provided an estimate of the relative discrimination ability when comparing scores. For this purpose, we use of a common comparator (CC) model (in our case the GOLD classification, since it is a variable supposed to be present in each COPD cohort); it constitutes a reference value for the performance of other scores, the value from which to subtract the possibly common biases [44].

Variance and covariance estimates for the ΔAUC values were estimated numerically using bootstrapping. We also confirmed consistency of bootstrapped variance estimates to those of the analytical formula for variances of paired differences in AUC [45] (results not shown).

Aggregated data on the cohort level for a cohort with k scores consist therefore of *k – 1* ΔAUC estimates and a corresponding *(k – 1) x (k – 1)* variance-covariance matrix.

To further clarify the methodology, we show the main steps with a small fictional example. Suppose we had 2 cohorts where score A and B could be evaluated (group 1: AB; cohorts P, R), 2 cohorts where A and C could be evaluated (group 2: AC; cohort S, T), 2 cohorts where A, B and D could be evaluated (group 3: ABD; cohorts U, V), and a final 2 cohorts where A, B, C, and D could be evaluated (group 4: ABCD, cohorts X and Y). Let us focus on group 3, constituted by the cohorts X and Y in which the scores A, B, and D can be used. We would obtain performance difference of the scores B and D in comparison to the score A for each of the cohorts, as reported in Table 1.

**Table 1:** Point estimate of the difference of AUC of the scores B and D with the score A in the group 3 of the fictional example

Cohort	ΔAUC – AB	ΔAUC – AC	ΔAUC – AD
X	0.05	–	0.07
Y	0.06	–	0.18

Analogously, in group 3, we would obtain variance-covariance matrices, like the ones reported in Table 2.

**Table 2:** Variance-covariance matrices of the difference of AUC of the scores B and D with the score A in the group 3 of the fictional example

Cohort X		
	0.0012	0.0005
	0.0005	0.0009
Cohort Y		
	0.0068	0.0051
	0.0051	0.0109

### Examination of network structure

Once the aggregated summary statistics were computed, we explored the structure of the network. In a first step, we divided the cohorts into groups based on which sets of scores could be evaluated.

**Fig. 1:**
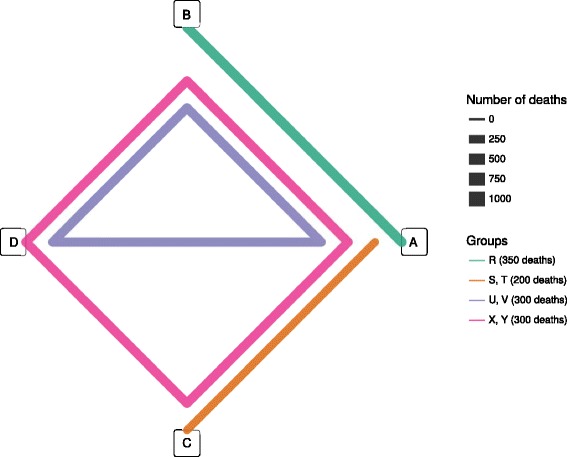
Network representation of the fictional example. **a**, **b**, **c**, **d** are the scores that are assessed in the fictional example

Each group is represented by a polygon, that passes by all the scores (i.e. the vertices) which can be evaluated in the cohorts constituting that group. The thickness of the polygon is directly proportional to the number of deaths in the group (Fig. 1).

Head-to-head comparisons within a group can be performed between any two scores connected in the same polygon.

For example, in group 4, A and D can be compared because they are both connected by the same polygon, even though there is no line directly connecting the two scores in that group.

According to Table 3, group 1, group 2, group 3 and group 4 have a cumulative number of 4000, 1000, 3000 and 2000 patients, respectively.

**Table 3:** Group characteristics of a fictional network (*g* identifies the group. *n* is the total number of subjects and *d* is the total number of deaths in each group. Additional characteristics are also listed: the *Q* statistic describing heterogeneity has *df* degrees of freedom, and τ gives the square root of the *τ* ^*2*^ statistics for between - cohort heterogeneity)

*g*	Scores	Cohorts	*n*	*d*	*Q*	*df*	*τ*
1	A, B	R	4000	350	28	0	0.019
2	A, C	S, T	1000	200	15.5	1	0.014
3	A, B, D	U, V	3000	300	13.4	2	0.004
4	A, B, C, D	X, Y	2000	300	9.6	3	0.036

### Multiple score comparison

The method of Lu et al. [35] was used to perform the multiple score comparison meta-analysis with Der Simonian-Laird random effects [46–49]. This method, which reinterprets frequentist NMA as a two-stage meta-regression model (using inverse variance weighted least squares estimation), was chosen as, compared to most of the network meta-analytic techniques, it can easily handle multi-score cohorts, and does not make unnecessary simplifications with respect to the correlations inherent in such trials, as discussed above. In the first stage, cohorts in which the same set of scores have been assessed are grouped together and meta-analysed separately.

An estimation *T* ^2^ of the between - cohort variance (*τ* ^2^) (i.e., the variance of the true performance difference across all studies) is the Der Simonian-Laird method [47] adapted to the network meta-analysis case [35]. Indeed, the *Q* statistic (adapted to network meta-analysis) is referred to a *χ* ^*2*^ distribution with degrees of freedom *df*_*g*_ *= (M*_*g*_ *− 1)(N*_*g*_ *− 1)*, where *M*_*g*_ is the number of scores compared in the group g and *N*_*g*_ is the number of cohorts belonging to the group *g*. Thus, the degrees of freedom are *df*_*1*_ = 1*0 = 0, df_*2*_ = 1*1 = 1, df_*3*_ = 2*1 = 2, df_*4*_ = 3*1 = 3. Table 3 allows us to calculate pooled *τ* ^*2*^ (according to the methods of moments) [46] with which we evaluate the weights used to obtain the weighted average of the performance estimate for the whole network (reported in the first 4 rows in Table 4).

**Table 4:** Stage I and Stage II results of the MSC meta-analysis in the fictional example of Table 1 (comparison with the A score)

Stage	*G*	B	C	D
I	1	0.09 (0.07, 0.12)		
I	2		0.18 (0.07, 0.29)	
I	3	0.10 (0.08, 0.12)		0.22 (−0.05, 0.50)
I	4	0.08 (0.04, 0..12)	0.15 (0.01, 0.29)	0.18 (0.05, 0.31)
II		0.09 (0.06, 0.13)	0.17 (0.10, 0.25)	0.21 (0.07, 0.35)

Analogously, extending the definitions from meta-analysis [46] to network meta-analysis [35] we calculate the variables C, Q and τ (τ represents the heterogeneity and deserves further discussion in the text later).

In Stage II the inverse variance weighted least square solution across all groups is found, thus we obtain the performance vector related to each score, best fitting the results of Stage I (for more details see Additional file 1). In the last row of Table 4, the final results of the MSC meta-analysis for the fictional example of are reported.

#### Transitivity, heterogeneity and inconsistency

The main assumptions to be met for performing a network meta-analysis are transitivity (a key assumption related to consistency), heterogeneity (differences in estimates of the same treatment or score contrasts coming from different studies) and inconsistency (comparing direct and indirect estimates, sometimes referred to as incoherence) [44, 50]. A key assumption of consistency is transitivity (sometimes referred to as similarity [51]) among the treatment effects [34, 44, 51–55], that is, that indirect comparisons are valid estimates of (unobserved) direct comparisons. Therefore one statistical approach to check for transitivity in our case is to explore the distribution variables giving case-mix across groups [56, 57], which we have adopted here using meta-regression. In practice, we used the definition of transitivity from a review paper on the topic [44] better matching our methodology, namely that the different sets cohorts do not differ with respect to the distribution of variables that could generate case mix variation. Thus, we evaluated by meta-regression analysis [58] the distribution of the variables that could generate case mix variation (like median and variability of age [25], range and variance of obstruction severity (i.e., FEV1% pred.), exercise capacity, size, mortality rate).

In case of variables directly affecting the performance, we used analysis of variance (ANOVA) to see whether the distribution of the identified variables was imbalanced in the groups and could consequently generate imbalance in the performance group by group. In case of homogenous groups, we cannot reject the null hypothesis of transitivity. With this method we assess as well, the eventuality that within-cohort heterogeneity could affect the analysis when “case-mix” is present (i.e. heterogeneity in the variables representing heterogeneity in the cohorts, like FEV%predicted range, that could affect the discriminative properties in the specific cohorts).

Heterogeneity could be described using a multivariate version of the usual *τ* ^*2*^ statistic, which in the Lu et al. [35] approach is considered on a group level at stage 1. They suggest that a pooled *τ* ^*2*^ may be a natural solution to situations where there are singleton groups (i.e. constituting only a cohort).

Inconsistency was primarily assessed visually by comparing direct and indirect comparisons from node-splitting side by side [59]. As a further check of inconsistency, we further examined the Q statistic (that is, the residual sum of squares) which can be used to reject the hypothesis of inconsistency between direct and indirect estimates if Q is greater than the χ^2^ statistic with N – K + 1 degrees at freedom at the 100(1 – α)% level [35], where N is the sum of the number of contrasts in each group, and K is total number of scores. Furthermore local consistency could be assessed at the group level by examining residuals and leverage statistics. Furthermore, we considered ways to calculate direct and indirect evidence within the network. Direct comparisons were computed by including only cohorts where both scores under consideration were present, and then performing the usual random effects meta-analysis [46]. However, defining loops of any order for indirect comparisons proved to be difficult in our setting, where the network is highly connected, and most cohorts have between 4 and 9 scores being assessed. Due to the various difficulties presented by studies with multiple scores, we chose to examine inconsistency in the network using “node-splitting” [59]. This approach avoids the need to define loops of any order, and includes all possible indirect evidence.

#### Consideration of missing data

The main analysis was performed without any imputation technique. A sensitivity analysis, using multiple imputation was also performed and it is shown in the online-only material. The results were not significantly different in the two cases.

#### COPD data description

Following the recommendation for large prospective studies [41], we based our analysis on a large-scale database (provided by the COPD Cohorts Collaborative International Assessment (3CIA) consortium [42]) from a diverse set of 24 cohort studies and 15,762 patients with COPD (1871 deaths and 42,203 person-years of follow-up). The cohorts were heterogeneous concerning geographic location, sample size, number of events and correspond to a broad spectrum of patients with COPD from primary, secondary and tertiary care settings. Mean FEV1 ranged from 30 to 70% of the predicted values, mean modified Medical Research Council (mMRC) dyspnea scores from 1.0 to 2.8 (the scale goes from 0 to 4, with 4 being the worst), mean number of exacerbations in the previous year (where available) from 0.2 to 1.7 and mean 6-min walk distance (where available) from 218 to 487 m. The follow-up period varied from cohort to cohort, thus we decided to use a minimum common time frame of 3 years. The mean age varies between 58 and 72 years. The outcome of interest was 3-year all-cause mortality. A table summarizing the clinical characteristics of the cohorts is reported in Additional file 1.

## Results

To illustrate an MSC meta-analysis, we compared the prognostic ability of various scores to predict mortality in patients with COPD. The COPD Cohorts Collaborative International Assessment (3CIA) [42] initiative contains individual data for around 16,000 patients (approx. 70,000 person years) with COPD from 26 cohorts in seven countries. Patients were considered to have COPD if the ratio of forced expiratory volume in 1 s (FEV1) to forced vital capacity (FVC) was less than 70%, regardless of the Global Initiative for Chronic Obstructive Lung Disease (GOLD) (2007) stage (I–IV) [60]. The minimum required set of variables for each cohort included vital status (up to death, loss to follow-up, or last data collection in June 2013), age, sex, pre-bronchodilator and post-bronchodilator FEV_1_ and dyspnoea MRC grade [42]. Most cohorts included many more variables allowing for the calculation of a total of 10 prognostic scores: GOLD (2007), GOLD (2011), updated ADO, BODE, updated BODE, eBODE, BODEx, DOSE, SAFE and optimised B-AE-D [7, 10, 61–65].

### Examination of network structure

We apply the MSC network meta-analysis of prognostic scores for 3-year mortality from the 3CIA data.

**Fig. 2:**
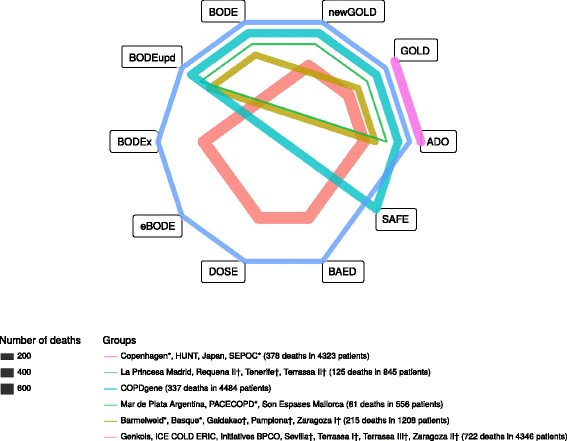
Depiction of network structure with lines weighted by the total number of deaths in the group. Abbreviations: GOLD, Global initiative for chronic Obstructive Lung Disease; BODE, Body mass index, airflow Obstruction, Dyspnoea and severe Exacerbations; BODE upd., BODE updated; ADO, Age, Dyspnoea, airflow Obstruction (we use the updated version of the ADO score in our analysis); e-BODE, severe acute exacerbation of COPD plus BODE; BODEx, Body mass index, airflow Obstruction, Dyspnoea, severe acute Exacerbation of COPD; DOSE, Dyspnoea, Obstruction, Smoking and Exacerbation frequency; SAFE, Saint George’s Respiratory Questionnaire (SGRQ) score, Air-Flow limitation and Exercise capacity; B-AE-D, Body-mass index, Acute Exacerbations, Dyspnoea. ^a^Cohorts belonging to the ADO or COCOMICS groups are marked with * or † respectively. ^b^The thickness of the lines is proportional to the number of deaths of the respective cohort. ^c^Below we report the composition of the group; each of them is identified by a specific color:  Copenhagen*, HUNT, Japan, SEPOC* (378 deaths in 4323 patients),  Barmelweid*, Basque*, Galdakao†, Pamplona†, Zaragoza I† (215 deaths in 1208 patients),  Mar de Plata Argentina, PACECOPD*, Son Espases Mallorca (61 deaths in 556 patients),  COPDgene (337 deaths in 4484 patients),  Genkols, ICE COLD ERIC, Initiatives BPCO, Sevilla†, Terrassa I†, Terrassa III†, Zaragoza II† (722 deaths in 4346 patients),  La Princesa Madrid, Requena II†, Tenerife†, Terrassa II† (125 deaths in 845 patients)

Based on the availability of the 10 scores in each cohort, the cohorts could be divided into 6 groups. The network structure is shown in Table 5 and in Fig. 2.

**Table 5:** Group characteristics of the network

g	Scores	Cohorts^a^	*n*	*d*	*Q*	*df*	*τ*
1	GOLD – ADO	Copenhagen*, HUNT, Japan, SEPOC*	4323	378	2.8	3	0
2	GOLD – ADO – BODE – BODEupd	Barmelweid*, Basque*, Galdakao†, Pamplona†, Zaragoza I†	1208	215	15.5	12	0.014
3	GOLD – GOLD (2011) – ADO – BODE – BODEupd	Mar de Plata Argentina, PACECOPD*, Son Espases Mallorca	556	61	10.9	8	0.025
4	GOLD – GOLD (2011) – ADO – BODE – BODEupd – SAFE	COPDgene	4484	337	7.46E-29	0	NA
5	GOLD – GOLD (2011) – ADO – BODEx – DOSE – BAED	Genkols, ICE COLD ERIC, Initiatives BPCO, Sevilla†, Terrassa I†, Terrassa III†, Zaragoza II†	4346	722	48.1	30	0.011
6	GOLD – GOLD (2011) – ADO – BODE – BODEupd – eBODE – BODEx – DOSE – BAED	La Princesa Madrid, Requena II†, Tenerife†, Terrassa II†	845	125	34.5	24	0.014

*Abbreviations*: *g* group, *n* number of subjects, *d* number of deaths, *Q* likelihood ratio statistic, *df* degrees of freedom, *τ* heterogeneity within the group, *GOLD* Global initiative for chronic Obstructive Lung Disease, *BODE* Body mass index, airflow Obstruction, Dyspnoea and severe Exacerbations, *BODE upd.* BODE updated, *ADO* Age, Dyspnoea, airflow Obstruction (we use in the our analysis the updated version of the ADO score), *e-BODE* severe acute exacerbation of COPD plus BODE, *BODEx* Body mass index, airflow Obstruction, Dyspnoea, severe acute Exacerbation of COPD, *DOSE* Dyspnoea, Obstruction, Smoking and Exacerbation frequency, *SAFE* Saint George’s Respiratory Questionnaire (SGRQ) score, Air-Flow limitation and Exercise capacity, *B-AE-D* Body-mass index, Acute Exacerbations, Dyspnoea

^a^Cohorts belonging to the ADO or COCOMICS groups are marked with * or † respectively. We notice that for group 4 the value of heterogeneity τau is not available (NA); indeed, that is a singleton group, where we cannot evaluate heterogeneity

Even if it would make sense to use absolute performance, we used relative performance of each score in comparison to a Common Comparator score, in order to get rid of possible common biases. We chose as Common Comparator score the GOLD classification. One cohort (COCOMICS Requena I) was excluded from the analysis because it only had sufficient variables to evaluate a single score (GOLD) and it would not contribute to the analysis. We had to further exclude the cohort A1ATD because there were no cases in the follow-up considered for our analysis (3 years) and the lack of events does not allow calculating an AUC. Of the remaining 24 cohorts, 4 had 2 scores (GOLD (2007), updated ADO), and the other 20 had between 3 and 9 scores assessed. We note that in no cohort all the 10 scores could be evaluated.

As GOLD (2007) is commonly used to classify the grade of severity of COPD patients, it could be assessed in all cohorts [60]. We note that direct evidence was available for 41 of 45 score comparisons, indirect evidence was available for other 16 comparisons (among which the four cases in which the direct comparison was missing).

### Multiple score comparison meta-analysis (MSC)

#### Transitivity, heterogeneity and inconsistency

To examine whether transitivity was fulfilled, we analysed the distribution of a number of possible variables potentially generating case-mix, following epidemiological reasoning and literature (age median and variability [25], FEV1 percent predicted range and variance, mortality percentage, exercise capacity range, number of events) across the groups using meta-regression. For the variables generating case-mix (whose meta-regression analysis were significant), the ANOVA analysis showed that the variables were balanced in the groups. Thus, we cannot reject the null hypothesis of transitivity.

Stage I group level results are presented in the top of Table 6, while the bottom rows show the stage II overall results from the network meta-analysis. GOLD (2007) scores ranged from 0.481 to 0.731, with a median of 0.614, and interquartile range (0.587, 0.641). Of the scores, the one that predicted mortality best was updated ADO with an average AUC 0.083 higher than that of GOLD (2007) (95% confidence interval: 0.069, 0.097), followed by the updated BODE which was associated with a 0.072 better AUC than GOLD (95% confidence interval: 0.051, 0.093) and eBODE (+0.069, 95% confidence interval: 0.044, 0.093). DOSE (+0.027, 95% confidence interval: 0.010, 0.045), optimised B-AE-D (+0.016, 95% confidence interval: −0.007, 0.038) and GOLD (2011) (+0.014, 95% confidence interval: 0.001, 0.028) and showed the worst performance in predicting mortality, only slightly better than GOLD (2007). The other scores, BODE, SAFE and BODEx showed moderate performance, between +0.045 and +0.064 improvement in AUC over GOLD.

**Table 6:** Stage I and Stage II results of the MSC meta-analysis (comparison with the GOLD classification)

Stage	g	ADO	BODEupd	eBODE	BODE	SAFE	BODEx	DOSE	BAED	newGOLD
I	1	0.097 (0.07, 0.123)								
I	2	0.098 (0.057, 0.139)	0.124 (0.078, 0.17)		0.098 (0.059, 0.137)					
I	3	0.044 (−0.03, 0.117)	0.023 (−0.054, 0.099)		0.019 (−0.054, 0.091)					−0.011 (−0.053, 0.03)
I	4	0.042 (0.01, 0.074)	0.043 (0.008, 0.078)		0.049 (0.017, 0.081)	0.037 (0.005, 0.069)				−0.008 (−0.038, 0.022)
I	5	0.099 (0.076, 0.123)					0.056 (0.035, 0.076)	0.036 (0.015, 0.057)	0.032 (0.005, 0.058)	0.028 (0.008, 0.047)
I	6	0.076 (0.027, 0.126)	0.043 (−0.006, 0.092)	0.048 (0.004, 0.093)	0.043 (0.001, 0.085)		0.030 (−0.015, 0.074)	0.021 (−0.023, 0.065)	−0.017 (−0.079, 0.045)	0.008 (−0.031, 0.047)
II		0.083 (0.069, 0.097)	0.072 (0.051, 0.093)	0.069 (0.044, 0.093)	0.064 (0.045, 0.082)	0.052 (0.022, 0.082)	0.045 (0.029, 0.061)	0.027 (0.010, 0.045)	0.016 (−0.007, 0.038)	0.014 (0.001, 0.028)

*Abbreviations*: *MSC* Multiple Score Comparison, *GOLD* Global initiative for chronic Obstructive Lung Disease, *BODE* Body mass index, airflow Obstruction, Dyspnoea and severe Exacerbations, *BODE upd.* BODE updated, *ADO* Age, Dyspnoea, airflow Obstruction (we use in our analysis the updated version of the ADO score), *e-BODE* severe acute exacerbation of COPD plus BODE, *BODEx* Body mass index, airflow Obstruction, Dyspnoea, severe acute Exacerbation of COPD, *DOSE* Dyspnoea, Obstruction, Smoking and Exacerbation frequency, *SAFE* Saint George’s Respiratory Questionnaire (SGRQ) score, Air-Flow limitation and Exercise capacity, *B-AE-D* Body-mass index, Acute Exacerbations, Dyspnoea

The first six rows show the Stage I results (group by group). The last row shows the Stage II results (namely the final results of the multiple score comparison meta-analysis)

The scores are ordered by performance of the prognostic scores in Stage II

Concerning heterogeneity, due to the group containing only a single cohort (group 6), we primarily considered a random effects analysis calculated using a pooled estimate of *τ* ^*2*^ for all groups, which was 0.00015, indicating a relatively low heterogeneity. The results of the MSC network meta-analysis were not substantially different when using the group-specific *τ* ^*2*^ estimates.

Possible inconsistency between direct and indirect comparisons was assessed using the *Q* statistic as described above. Overall, Q for the random effects analysis was 22.1 with 16 degrees of freedom. Keeping in mind that in this case (as in classical network meta-analysis) the inconsistency test has low power, since *Q* was smaller than the corresponding χ^2^ statistic of 26.3, we do not reject the hypothesis of consistency (*P* = 0.14).

Both direct and indirect estimates of the score comparisons were calculated using node-splitting [59], and compared visually. The results are similar to each other and to the estimates provided by the network meta-analysis (see Additional file 1 for further discussion).

#### Consideration of missing data

As a secondary analysis, the entire meta-analysis was repeated in a multiple imputation framework, as described above. The results were similar to the main analysis without imputation (see Additional file 1) [1, 66, 67].

## Discussion

To the best of our knowledge, the MSC meta-analysis proposed in this paper represents the first methodology to evaluate the comparative prognostic properties of prediction models that synthesizes all available (direct and indirect) evidence. The application of the MSC meta-analysis could provide different clinical fields with a clear indication of which is the best-performing prediction model, paving the way for a standardized clinical application. While there are a number of issues when adapting usual NMA methodology to MSC, they can be addressed in a straightforward manner. Multi-score studies are considered in our approach by explicitly using covariance estimates for the various prognostic scores. Calculation of such estimates using bootstrapping may be computationally intensive but is not difficult to implement. The approach presented here can be used to compute prognostic score comparisons for the entire network of evidence, as well as both direct and indirect comparisons between scores.

Despite these adaptations, the results of the MSC meta-analysis are clear, and may be interpreted in a fashion similar to standard network meta-analysis results. The only difference is that the performance measure is not mean difference between treatments, or log odds ratio, but difference in performance measure such as AUC. Measures of heterogeneity and inconsistency can however be calculated and interpreted in the usual fashion [44]. For instance, a definition similar to the one used for the heterogeneity for meta-analysis of direct comparisons, can be used for the heterogeneity of network meta-analysis, adapting a definition used for multi-arm trials to multiple score comparison. Since we have singleton groups in our MSC data (group 6 in our database), it is recommended in our case to use pooled estimate of the *τ* ^*2*^ (*τ* ^*2*^_*pooled*_) [35], i.e. a multivariate version of the pooled estimate for the heterogeneity variance (more technical details are provided in Additional file 1).

We used one of the scores as a common comparator, which would not generally be necessary, but may be easily possible in this MSC setting.

Performance of the considered prognostic scores can be computed from the individual patient data (IPD) directly; this is how we approach the problems having at our disposal a large-scale IPD database. The group results (Stage I) arise from averaging the cohort results, that, in turn, are calculated using the IPD of each cohort. If no IPD are available, instead, there are two possibilities: use published results, or send cohorts code to extract the aggregated performance measures individually. Use of published results requires that comparisons have been reported for more than one score, which in practice may almost never be the case. Sending code to obtain aggregated measures may be an optimal approach in cases where no large-scale collaboration exists, and published results are not detailed enough.

We used all-cause mortality as outcome. Apart from being clinically relevant, mortality is the easiest outcome that we can expect to evaluate in a cohort, with a hard definition. This makes it easier to reduce the problems related to miss-classification or missingness of the outcome [68, 69].

Given the patterns of missing data (in general, the variables are completely or almost completely missing or not missing at all) a sensitivity analysis performed after multiple imputation is providing similar results to the analysis without imputation (a comparison is provided in Additional file 1). Analogously, a sensitivity analysis using heterogeneity group by group gives similar results than using a pooled heterogeneity (here recommended because of the network structure; more details are available in Additional file 1).

There are a few limitations to this approach to MSC. Although the analysis can be implemented as outlined by Lu et al. [35] (Additional file 1), creating an input dataset in a spreadsheet may be less than straightforward. We have therefore provided example R code to convert a dataset of prognostic scores to a MSC meta-analysis, without first making a table of cohort-level summary statistics, as is often performed. We note however that such a dataset including a column for each cell of the variance-covariance matrices could be analysed using mvmeta in Stata. Creating that kind of summary dataset might be useful to go along with the network meta set of commands in Stata [70]. We focused on implementing this approach starting from the raw prognostic scores from individual patients, which had been calculated using raw data from the international collaboration [42].

## Conclusions

In summary, we have adapted methodology from network meta-analysis to compare prognostic scores from individual patient data across different cohorts. This approach permits concurrent external validation of the scores in a consistent analysis explicitly including correlations between the scores on a cohort level. Estimates of differences in performance can be estimated for the entire network, as well as for both direct and indirect comparisons of scores. Results of the MSC meta-analysis can be interpreted in a manner similar to that of the usual network meta-analysis, regardless of the performance measure used. Our application to prognostic scores showed that the ADO and updated BODE scores have the best discriminative performance to predict mortality for patients with COPD. The meta-analysis could also be repeated for a number of different performance measures in order to describe multiple facets of the prognostic scores (e.g. discrimination and calibration [1]) or using reclassification methods (like the net reclassification index, NRI [71]) or to aid in the interpretation of the results. Development of clearer data input formats as well as more automated would provide opportunities for further methodological research in MSC meta-analysis.

## Additional file

Additional file 1: Multiple Score Comparison: A network meta-analysis approach to comparison and external validation of prognostic scores. Additional statistical background is provided, as well as further details on heterogeneity, transitivity, inconsistency, indirect evidence, and multiple imputation, as well as a table summarizing the scores compared. (DOCX 750 kb)

## Abbreviations

COPD: Chronic obstructive pulmonary disease
IPD: Individual patient data
MSC: Multiple score comparison
