# Correction to: Multiple Score Comparison: a network meta-analysis approach to comparison and external validation of prognostic scores

**DOI:** 10.1186/s12874-018-0481-2

**Published:** 2018-02-12

**Authors:** Sarah R. Haile, Beniamino Guerra, Joan B. Soriano, Milo A. Puhan

**Affiliations:** 1 Epidemiology, Biostatistics and Prevention Institute, University of Zurich, Zurich, Switzerland; 2 Servicio de Neumología, Instituto de Investigación del Hospital Universitario de la Princesa (IISP), Universidad Autónoma de Madrid, Madrid, Spain; 3 Epidemiology & Department of Epidemiology, Johns Hopkins Bloomberg School of Public Health, Baltimore, USA

## Correction

Following publication of the original article [1], a member of the writing group reported that his name is misspelt. The paper should appear in Pubmed under “Ter Riet G”, not as “Riet GT”.
