# Supplementary material for: Multiple Score Comparison: a network meta-analysis approach to comparison and external validation of prognostic scores
Source: BMC Med Res Methodol. 2017 Dec 21;17:172. doi: 10.1186/s12874-017-0433-2 (PMC5740913; doi:10.1186/s12874-017-0433-2)
Supplement: Additional file 1: — Multiple Score Comparison: A network meta-analysis approach to comparison and external validation of prognostic scores. Additional statistical background is provided, as well as further details on heterogeneity, transitivity, inconsistency, indirect evidence, and multiple imputation, as well as a table summarizing the scores compared. (DOCX 750 kb) [file 12874_2017_433_MOESM1_ESM.docx]

Multiple Score Comparison: A network meta-analysis approach to comparison and external validation of prognostic scores

**Online-Only Material**

**Table of contents**

STATISTICAL BACKGROUND 2

Code discussion 7

Data Description 9

Examination of network structure 12

R Code for Network Plot 14

MSC: HETEROGENEITY 21

Tau pooled vs tau group 21

MSC: TRANSITIVITY 25

AUC vs FEV1 %pred. range 27

AUC vs FEV1% pred. variance 30

AUC vs mortality percentage 32

AUC vs exercise capacity range 34

AUC vs median age 36

AUC vs variance age 38

AUC vs size cohort (#cases) 39

INCOSISTENCY 40

INDIRECT EVIDENCE 42

MULTIPLE IMPUTATION 43

SUCRA p-score 46

CORRELATIONS AMONG SCORES 47

SCORES 47

REFERENCES 48

# STATISTICAL BACKGROUND

In this section we present the statistical background on which we relied to develop the MSC meta-analysis, mainly related to which performance measure to use, how to take advantage of indirect comparison through network meta-analysis.

The first task was to determine an appropriate endpoint for comparison of prediction models. The chosen performance measure should be a single value, should have a scale independent of the data considered, should not be dependent on any grouping, and should have a defined variance estimate. We considered area under the ROC curve (AUC), Brier score, the Hosmer-Lemeshow test [1–3], the calibration slope[4] and the net reclassification index (NRI)[5]. The scale of the Brier score depends on the data and the score has no meaningful variance estimate. The Hosmer-Lemeshow test is highly dependent on the grouping used[2, 6], and was therefore also excluded. NRI has defined scales and variance estimates (Pencina 2008). However, NRI has been shown not to be a proper score in that even an uninformative new variable can lead to significant improvement in NRI (Pepe 2015) and some authors have suggested that it may give misleading results. The calibration slope has a defined variance estimate, and is represented by a beta coefficient from a regression model; thus, has maximum likelihood estimation (MLE) properties (e.g. approximate normality).(Snell 2016) However, the calibration slope has of difficult interpretation in a comparative setting because the optimal value of 1 is not an extreme of its range Furthermore, its trend would make it difficult to give a meaning to difference of performance and then to eventually rank the scores. Finally, the AUC has a set range of values regardless of the cohort to which it is applied [0,1], it is easy to provide a ranking with its values (the higher the better the model), it has an analytical form its variance (Hanley 1982), it is easy to interpret, insensitive to outcome incidence and commonly accepted by the prediction model community.

Therefore, we selected AUC for our methodology. AUC gives an estimate of a prediction model’s discriminative ability, [7] and therefore we consider differences in AUC, as a comparative measure of model performance. In principle, ratios of AUCs could have also be considered but in the literature the difference of AUCs has received more attention. Variance estimates for differences in AUC can be estimated[8, 9] .

The next step was to examine the features of the network, and find an appropriate method for performing the MSC. Already considering the difference in performance between two scores evaluated on the same patients a correlation is introduced(Hanley 1983). A guide was recently published to highlight the correct procedure for an overview of the performance of a score by performing a systematic review and meta-analysis across studies.[10] However, in general, more than 2 scores per cohort can be assessed. Franchini et al[11] have shown that incorrect results can be obtained if the correlation in multi-arm trials is ignored. Simple meta-analysis of prognostic scores was performed for the comparison of 2 scores (one obtained from the other adding a biomarker, thus, for performance measure the Net Reclassification Index was used).[12] We extend this approach (by MSC meta-analysis) to multiple comparisons. We therefore needed to critically consider how correlations between performance measures within a cohort are incorporated into the network meta-analysis.

Consider a meta-analysis comparing 3 scores A, B and C. If we assume that the variance is same for each score comparison, say τ^2^/2^[^^13]^, then the covariance of any two contrasts is just. Salanti et al. 2008 notes that this assumption may be “difficult to defend.” Franchini et al. 2012 (Appendix B) have shown that if the same control arm is used, say A for contrasts AB and AC, and if treatment arms A, B and C are mutually independent, then the covariance between AB and AC is equal to the variance of A. While Franchini et al.’s assumption of independence of treatment arms is appropriate in an MTC setting, there are no randomized independent treatment arms when performing multiple score comparison. Rather, MSC assesses the performance of each score in a cohort on the same set of patients, leading to two sources of dependence in MSC: 1) using a common comparator, and 2) the correlated structure of the estimated performance measures. Therefore, neither of the two approaches to modelling the correlation in multi-arm trials is appropriate in an MSC setting.

Ideally, it would be possible to estimate the covariance between two comparisons of prognostic scores directly from the data, and plug these estimates into the model. For the first of these tasks, explicit variance and covariance formulas are required. While explicit formulas exist for the variance of the difference of two AUC estimates [8, 14], this is not the case for the covariance of two (or more) differences in AUC. In an MSC meta-analysis using individual patient data or code sent to investigators to compute aggregate performance measures based on their individual patient data, bootstrapping[15] could be used within each cohort to estimate the variance of each score comparison, as well as the covariance between score comparisons. Indeed, for score performance comparison formulas for two-score comparison exist (Hanley 1983), but for cases with more scores the easiest way to obtain performance comparison and co-variance matrix of the performance is bootstrapping.(Snell 2016, Riley 2015) In this way it would be possible to explicitly estimate the entire variance-covariance matrix for the score comparisons in each cohort without requiring a formula. This strategy would also work for other performance measures. For an MSC meta-analysis based on published results, assumptions such as those described above may need to be made about the correlation structure.

For the second task of explicitly including the correlation structure, we needed a statistical approach to meta-analysis that made no assumptions about the correlation structure in multi-score studies. Methodology work on network meta-analysis has increased in recent years, leading to wide range of possible statistical approaches [13, 16–22]. Sutton & Higgins 2008 and Efthimiou et al. 2016 provide an overview of current methodology. Many approaches assume common variances among treatment arms in the same trial [13], implying that the correlation between arms of a multi-arm trial is 0.5, though some permit heterogeneity of variance [16]. Bayesian models are often preferred because of the complexity of the data structure, although even those have been deemed “cumbersome” if not all trials have the same number of treatment arms [22, 25], and require assigning prior distributions to all parameters in the meta-analysis model. Lu et al. 2011 proposed one frequentist formulation of network meta-analysis methods, which describes MTC as a two-stage procedure, elucidating the statistical properties of an existing network meta-analysis methodology.(Gleser & Olkin 1994) Their approach shows that network meta-analysis is like linear regression, and includes multi-arm trials of any size explicitly without making further assumption about correlation structures. The Lu-Ades [26] model has also been shown to be equivalent to the design-by-treatment interaction model [27–29] but can be programmed and interpreted in the frequentist fashion. Kessels et al. 2013 have also discussed the issue of correlation in multi-arm trials explicitly and proposed simple solutions to it. Unfortunately their method was not directly applicable to the MSC setting, due to the presence of cohorts with more than 2 scores.

Another important task in performing network meta-analysis is to understand if and where inconsistency between direct and indirect estimates of treatment effects occurs Dias et al. 2010 describe two different approaches to examine inconsistency in MTC. The first approach, “back calculation” [32] uses the loop structure in the network to compare the direct estimate with its indirect counterpart as in the consistency equation above, and works assuming no indirect evidence from multi-arm trials is present. The second approach, “node-splitting”. ‘Node-splitting’ separates evidence on a particular comparison (node) into ‘direct’ and ‘indirect’. Thus, in our MSC meta-analysis, the evaluation of the performance comparison between two scores (say A and B) Δ_AB_ is decomposed into Δ^dir^_AB and_ Δ^ind^_AB_ (respectively the direct and indirect component). In practice, in multi-score cohorts the two relevant scores are included in the estimation of Δ^dir^_AB_, while the remaining scores are included in the indirect estimation of the relevant performance comparison through the MSC meta-analysis comparison (Δ^ind^_AB_). Even if the scores A and B are not directly compared, they are in general present either one or the other in some cohorts and, thus, their indirect comparison can be performed anyway. Multi-arm trials can be split in a similar fashion, so that those treatment arms which do not contribute to the direct estimate are included in the calculation of the indirect estimate. In this manner, all data in the meta-analysis contributes to either the direct or the indirect evidence, but not both.

Another important aspect of this MSC meta-analysis was how to depict the network of evidence graphically. Some authors have used polygons to show multi-arm trials, for example in the smoking cessation example. Recently, Rücker et al. 2016 proposed an automated method for drawing such network graphs, and suggested using filled polygons to represent multi-arm trials.

## Code discussion

The two stage method also has a significant drawback in that it cannot be implemented using standard regression models if any multi-arm trials are present. Lu et al. 2011 provide R code [34] which could be adapted for any number of treatment arms or scores, and suggested that Stata’s [35] function mvmeta[36] could also be used to perform meta-analysis for any number of arms. Both of these implementations unfortunately have their own disadvantages. The R version requires inputting a variance-covariance matrix for each cohort, making a spreadsheet an inappropriate tool to store the cohort level aggregate data. On the other hand, the mvmeta function in Stata requires one row per study as expected, as well as 1 column for each treatment contrast, 1 column for each variance estimate, and additional columns for each of the covariance estimates. Such a data table may be easy to understand, but it would be unwieldy even with a relatively small number of scores. For example, five prognostic scores require 4 columns for performance comparisons, 4 for variance estimates, and another 10 for the covariance estimates (total 18), while for 9 we need a total of 44. Furthermore, the network meta and mvmeta Stat commands make specific assumptions (off-diagonal entries of the covariance matrix set to 0.5, assumption difficult to defend in our case, Salanti 2008) on the correlation structure (the provided options did not look like ready yet for an extension to our methodology, even if we realise, with a little work, Stata can perform the same analysis)(White 2015).

## Data Description

In T**able** 1 Study characteristics the data related to the main variables in the cohorts are summarized.

| T**able** 1 Study characteristics | | | | | | | | | | | | | | |
| --- | --- | --- | --- | --- | --- | --- | --- | --- | --- | --- | --- | --- | --- | --- |
| Variable  Cohort | # Events | # patients | Person-years | Mean age: years | Men: % | Mean FEV1%  pred. | Mean mMRC | Past exacerbators: % | Mean # prev. exacerbations | Current Smoker: % | Mean BMI | Mean 6MWT: meters | Mean SGRQ | Mean CAT |
| COPDgene | 337 | 4484 | 10603 | 63 (9) | 56 | 57.4 (22.8) | 1.5 | 0.16 |  | 43 | 27.9 (6.1) | 376.1 (124.1) | 36.9 (22.9) |  |
| Sevilla^a^ | 205 | 596 | 1562 | 66 (10) | 95 | 43.5 (13.3) | 1 | 0.25 | 1.16 | 24 | 29.2 (5.7) |  |  |  |
| Copenhagen^b^ | 186 | 2287 | 6618 | 61 (9) | 54 | 70.5 (23.7) | 1.3 |  |  | 71 | 25 (4.2) |  |  |  |
| Genkols | 126 | 954 | 2708 | 65 (10) | 61 | 46.9 (17) | 1.3 | 0.15 | 0.6 | 47 | 25.4 (5) |  |  |  |
| Zaragoza II^a^ | 118 | 1150 | 3069 | 63 (9) | 93 | 62.3 (20.3) | 1.1 | 0.17 | 0.91 | 34 | 27.5 (4.8) | 356.2 (153.7) |  |  |
| HUNT | 116 | 1571 | 4583 | 63 (13) | 62 | 63.8 (18.7) | 1.3 |  |  | 47 | 26.4 (4.4) |  |  |  |
| Galdakao^a^ | 92 | 543 | 1497 | 68 (8) | 96 | 55 (13.3) | 0.9 | 0 | 0.65 | 21 | 28.3 (4.4) | 408.9 (92.4) |  |  |
| Barmelweid^b^ | 79 | 232 | 555 | 72 (9) | 60 | 45.2 (16.1) | 1.1 |  |  | 21 | 26 (6.3) | 363.4 (126.8) |  |  |
| Terrassa III^a^ | 78 | 181 | 423 | 72 (10) | 95 | 45.2 (14.4) | 1.2 | 0.31 | 1.28 | 23 | 27.9 (5) | 330.4 (105.8) |  |  |
| Initiatives BPCO | 76 | 930 | 1525 | 64 (10) | 77 | 52.4 (20.3) | 1.1 | 0.4 | 1.65 | 28 | 25.4 (5.5) | 387.4 (120.8) | 43.9 (19) |  |
| Terrassa I^a^ | 72 | 135 | 284 | 72 (9) | 92 | 41.3 (13) | 1.3 | 0.25 | 1.03 | 17 | 26.3 (4.9) |  |  |  |
| SEPOC^b^ | 61 | 318 | 871 | 65 (9) | 100 | 45 (18.3) | 1.5 |  |  | 38 | 26.4 (4.2) |  |  |  |
| Requena II^a^ | 52 | 186 | 396 | 71 (9) | 99 | 44.5 (16.5) | 1 | 0.16 | 0.62 | 17 | 28.1 (5.2) | 380.1 (111.9) |  |  |
| ICE COLD ERIC | 47 | 400 | 1071 | 67 (10) | 57 | 55.3 (16.5) | 1.5 | 0.13 | 0.58 | 39 | 26.1 (5.2) |  |  |  |
| PAC-COPD^b^ | 41 | 342 | 980 | 68 (9) | 93 | 52.4 (16.2) | 1 | 0.04 |  | 33 | 28.2 (4.7) | 435.5 (90.6) |  |  |
| Tenerife ^a^ | 34 | 275 | 653 | 63 (10) | 79 | 55.8 (21.2) | 1.2 | 0.06 | 0.37 | 42 | 27.3 (5.1) | 487.4 (87.5) |  |  |
| Terrassa II^a^ | 28 | 66 | 145 | 72 (9) | 98 | 30.2 (12.9) | 1 | 0.42 | 1.81 | 14 | 25.7 (4.3) | 217.7 (76.6) |  |  |
| Requena I^a^ | 23 | 174 | 393 | 72 (9) | 99 | 48.1 (16.8) | 1.2^c^ | 0.03 | 0.22 | 23 | 28 (4.2) | 434.4 (125.3) |  |  |
| Zaragoza I^a^ | 21 | 137 | 379 | 66 (8) | 99 | 49.8 (17.6) | 1.1 |  |  | 27 | 27.7 (4.6) | 449 (91.9) |  |  |
| Son Espases Mallorca | 17 | 115 | 292 | 70 (7) | 79 | 41.5 (13.4) | 1 | 0.59 |  | 27 | 27.1 (5.9) | 401.5 (89.7) |  | 16.6 (8.2) |
| Basque^b^ | 16 | 106 | 299 | 71 (9) | 98 | 46.9 (11.4) | 0.6 |  |  | 23 | 26.1 (4.9) | 442.9 (95.4) |  |  |
| Japan | 15 | 147 | 409 | 69 (7) | 100 | 47.1 (17.5) | 0.9 |  |  | 22 | 21 (2.9) |  | 36.6 (16.5) |  |
| La Princesa Madrid | 11 | 318 | 633 | 71 (10) | 74 | 50 (19.8) | 1.1 | 0.18 | 0.77 | 19 | 26.2 (5.1) | 337.1 (92.8) |  |  |
| Pamplona^a^ | 7 | 190 | 470 | 65 (8) | 84 | 68.9 (19.9) | 1.1 |  |  | 37 | 27 (4.4) | 463.2 (113.9) |  |  |
| Mar de Plata Argentina | 3 | 99 | 147 | 64 (9) | 60 | 48.8 (18.6) | 1 | 0.29 |  | 21 | 27 (5.6) | 353.2 (128.7) |  | 16.1 (7.8) |
| A1ATD^d^ | 0 | 308 | 834 | 58 (10) | 60 | 53.1 (25.1) | 1.2 | 0.52 |  | 5 | 25.7 (4.9) |  | 50.8 (19.9) | 20.5 (8.1) |
| **Abbreviations: FEV1% pred.=forced expiratory volume in 1 second percentage predicted; mMRC=modified Medical Research Council (MMRC) dyspnea scale; past exacerbators are defined as patients with more than 1 exacerbation in the previous year; mean # previous exacerbations are referred to the previous year; BMI=body-mass index; 6MWT=6-minute walk test; SGRQ=Saint George’s Respiratory Questionnaire; CAT=COPD Assessment Test.**  **The cohorts are presented in decreasing order of number of events. Most of the variables available provided by the 3CIA collaboration for the different cohorts are shown. In particular, we show all the variables constituting the scores analyzed in our study. We present the standard deviation for all individual variables, whose distribution is approximately normal; this is not the case for count (with small numbers) or categorical variables, like number of previous exacerbations or mMRC).**  **^a^Cohorts belonging to the COCOMICS collaboration**  **^b^Cohorts belonging to the ADO collaboration. For information concerning the cohorts, please see** [37]**.**  **^c^ Since none of the score could be evaluated in the cohort “Requena I” (mainly because the variable dyspnoea was missing for 95% of the patients, i.e. for 165 out of 174patients, please see supplementary material), this cohort was excluded from the analysis.**  **^d^ Since there was no event in a follow-up of 3 years, the cohort “A1ATD” was excluded from the analysis.**  **Missing cells correspond to variables that are completely missing in the cohort of the correspondent row.** | | | | | | | | | | | | | | |

## Examination of network structure

Drawing the network plot to show this structure graphically proved to be an unexpectedly difficult task. Standard software packages (e.g. netmeta in R [33]) tend to show all possible pairwise comparisons even in multi-arm trials. This is reasonable for 3-arm trials [38–40], but quickly obscures the network structure with a larger number of arms, a known problem with “dense” networks [41]. Another concern is that those network plots would indicate how often scores are concurrently evaluated, but obscure in which cohorts or groups they were evaluated; this information is of great value in MSC meta-analysis, where the number of concurrently evaluated scores could easily be quite high (differently from MTC). Thus, it was almost impossible to discern the structure of the network from the plot alone.

Each group is represented by a polygon, that passes by all the scores (i.e. the vertices) which can be evaluated in the cohorts constituting that group. The thickness of the polygon is directly proportional to the number of deaths in the group. Head-to-head comparisons within a group can be performed between any two scores connected in the same polygon. Code to produce a network plot using the R package ggplot2 [42] has been provided below.


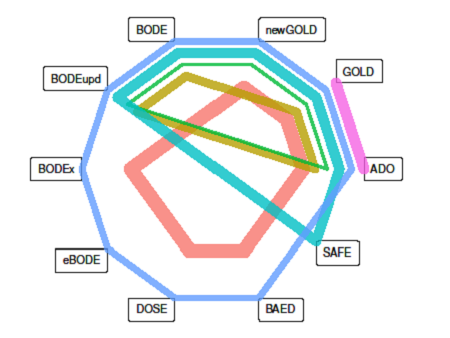


Figure 1. Depiction of network structure with lines weighted by the total number of deaths in the group

Abbreviations: GOLD, Global initiative for chronic Obstructive Lung Disease; BODE, Body mass index, airflow Obstruction, Dyspnoea and severe Exacerbations; BODE upd., BODE updated; ADO, Age, Dyspnoea, airflow Obstruction (we use the updated version of the ADO score in our analysis); e-BODE, severe acute exacerbation of COPD plus BODE; BODEx, Body mass index, airflow Obstruction, Dyspnoea, severe acute Exacerbation of COPD; DOSE, Dyspnoea, Obstruction, Smoking and Exacerbation frequency; SAFE, Saint George’s Respiratory Questionnaire (SGRQ) score, Air-Flow limitation and Exercise capacity; B-AE-D, Body-mass index, Acute Exacerbations, Dyspnoea. This figure is reported in the supplementary material as well, in order to make clearer the network depiction strategy.

^a^Cohorts belonging to the ADO or COCOMICS groups are marked with * or † respectively.

^b^The thickness of the lines is proportional to the number of deaths of the respective cohort.

^c^Below we report the composition of the group; each of them is identified by a specific color:

Copenhagen*, HUNT, Japan - SEPOC* (378 deaths in 4323 patients)

Barmelweid*, Basque*, Galdakao†, Pamplona†, Zaragoza I† (215 deaths in 1208 patients)

Mar de Plata Argentina, PACECOPD*, Son Espases Mallorca (61 deaths in 556 patients)

COPDgene (337 deaths in 4484 patients)

Genkols, ICE COLD ERIC, Initiatives BPCO, Sevilla†, Terrassa I†, Terrassa III†, Zaragoza II† (722 deaths in 4346 patients)

La Princesa Madrid, Requena II†, Tenerife†, Terrassa II† (125 deaths in 845 patients)

Depicting the network graphically in order to better visualize its features was a more difficult task than expected. Our approach uses unfilled polygons of differing sizes to clearly show combinations of prognostic scores occurring together in the same cohort or group of cohorts, this comes at the expense of downplaying all individual head-to-head comparisons could be made.

## R Code for Network Plot

library(ggplot2)

datsumm <- data.frame(g = c("A", "A-C", "A-C-D-E", "A-B-C", "A-B-C-D-E",

"A-B-C-D-E-F-G-H",

"A-B-C-D-E-I", "A-B-C-G-H"),

n = c(162, 4313, 1195, 717, 556, 826, 4484, 3667))

allg <- sort(unique(unlist(strsplit(as.character(datsumm$g), "-"))))

## draw the points on a unit circle

angles <- seq(0, 2 * pi, length.out = length(allg) + 1)

angles <- angles[angles != 2*pi]

x <- cos(angles)

y <- sin(angles)

# reorder summary dataset (this rearranges the groups in the plot, because the first listed is the innermost drawn, and so on out to the last listed being the outermost, or however you wish)

#datsumm <- datsumm[8:1, ]

loops <- NULL

for(i in 1:nrow(g)){

this.g <- which(allg %in% unlist(strsplit(as.character(datsumm$g[i]), "-")))

r <- i / 2 + nrow(datsumm)/2

this.loop <- data.frame(cohorts = i, x = r * x[this.g], y = r * y[this.g],

nms = allg[this.g], n = datsumm$n[i], deaths = datsumm$d[i])

loops <- rbind(loops, this.loop)

}

loops$cohorts <- factor(loops$cohorts)

loops$nms <- as.character(loops$nms)

loops$lastone <- as.numeric(!duplicated(loops$nms, fromLast = TRUE))

ggplot(data = loops, aes(x, y)) +

geom_point() +

geom_label(aes(label = nms),

data = subset(loops, lastone == 1),

vjust = "outward", hjust = "outward") +

geom_polygon(aes(color = factor(cohorts),

size = n), fill = NA) +

coord_equal() + guides(fill = FALSE, color = FALSE, size = FALSE) +

xlim(-10, 10) + ylim(-10, 10) + theme_void()

#### Multiple score comparison (MSC) meta-analysis by two-stage approach [26]

The use of mixed treatment comparison (MTC) to compare all trials in an area is starting to be recognized by prominent general medical journals [43]. We will adapt a methodology from multiple treatment comparison network meta-analysis [26] to concurrently externally validate and compare prognostic scores from individual patient data across different cohorts, explicitly including correlations [11] between the scores on a cohort level.

We will also re-interpret MTC as a two stage process:

1. Ordinary meta-analysis to gain the direct estimates for corresponding pooled effect estimates
2. Based on the direct estimates and their variances from the first stage, they obtain to find the optimal estimate of the pooled effect parameters that obeys the fundamental consistency equations

##### **Stage I. Indirect comparisons meta-analysis**

Cohorts at our disposal are classified into “groups” according to which scores it is possible to evaluate by their data. Each of the G groups will be indicated as *S_g_* (with g=1,…,G). *N_g_* will be the number of studies in the category *S_g_*; *M_g_* is the number of scores (m_g1_ m_g2_, m_gMg_) that is possible to evaluate in the group *S_g_* (cohorts in which less than 2 scores can be evaluated will be excluded). We will refer to the generic cohort represented by the group Sg, in which *N_g_* scores can be evaluated, as a *N_g_*-score group. A hypothetical representation of the groups follows:

| *Group* | No. of cohorts | Scores |
| --- | --- | --- |
| *S_1_* | 20 | *X, Y* |
| *S_2_* | 16 | *Y, Z* |
| *S_3_* | 8 | *X, Y, Z* |
| *…* | … | … |
| *S_g_* | *N_g_* | m_g,1_, m_g,2_,…,m_g,Mg_ |
| *…* | … | … |
| *S_G_* | 1 | m_g,1_, m_g,2_,…, m_G,MG_ |

Table 2. Groups of scores that can be evaluated with labels of the scores and the number of studies in which that combination of scores can be evaluated

Looking at the hypothetical Table 2, the groups S_1_ and S_2_ represent a direct comparison of the scores *X* and *Y*, i.e., they are two-score groups. To these groups belong, respectively, 20 (N_1_) and 16 (N_2_) cohorts. That means that, respectively, 20 and 16 cohorts allow the evaluation of the score *X* and *Y*, and *Y* and *Z*, respectively. S_3_ represents a group in which the scores *X*, *Y* and *Z* can be compared in 8 (i.e., M_3_=8) different cohorts, thus S_3_ is a three-score group.

The objective of the first stage meta‐analysis is to obtain the pooled performance difference estimate $\Delta$_g_ and its variance V_g_≡W^−1^_g_ for each score group g, where Δ_g_ and V_g_ are scalars for those two-score comparison groups *S_g_* (i.e., in which M_g_=2), while they are vectors (that we will than indicate with bold $\boldsymbol{\Delta}$**_g_** and **W^−1^_g_**), if *S_g_* is a n-score comparison group with n bigger than 2 (i.e., in which M_g_>2); **Δ_g_** and **W^−1^_g_** will be in general a (M_g_ -1) vector and a (M_g_ -1)* (M_g_ -1) covariance matrix. Thus, at the end of the first stage, we will have a collection of summarized data {(**Δ_g_**, **W^−1^_g_**), g=1,…, G}, that include the direct evidence about all available comparisons.

Several methods to estimate the pooled effect in ordinary meta-analysis for two-arm trials (in our case, two-score studies) exist [44]. Less common are multi-arm trials approaches. For instance, concerning a three-score group (*M_g,j_* = 3), we can refer to the estimate vector $\boldsymbol{\Delta}_{j,g}$ regarding the individual *j-th* cohort belonging to *S_g_*, as

|  | $\boldsymbol{\Delta}_{\boldsymbol{jg}}={(\Delta_{jXY}, \Delta_{jXZ})}^{T}$*,* | (1) |
| --- | --- | --- |
|  |  |  |

Where Δ*_jXZ_ ,* represents the performance difference related to the score *X* and *Y* evaluated in the cohort *j* and such that

The terms $\Delta_{jXY}$ and $\Delta_{jXZ}$ are evaluated as follows:

|  | $\Delta_{jYZ}={\left( -1,+1 \right)\boldsymbol{\Delta}}_{\boldsymbol{jg}}=\Delta_{jXZ}-\Delta_{jXY}$ | *(2)* |
| --- | --- | --- |
|  |  |  |

The expression for the covariance matrix is:

|  | $\boldsymbol{\Delta}_{jg}=\left( \begin{aligned} \Delta_{jXY} \\ \Delta_{jXZ} \end{aligned} \right)=\left( \begin{aligned} \Delta_{jY}- \Delta_{jX} \\ \Delta_{jZ}-\Delta_{jX} \end{aligned} \right)$*.* | *(3)* |
| --- | --- | --- |
|  |  |  |

|  | ${\boldsymbol{W}_{\boldsymbol{jg}}}^{\boldsymbol{-1}}=var\left( \Delta_{\boldsymbol{jg}} \right)=\left( {{var(\Delta}_{jXY}) \atop{cov(\Delta}_{jXY,XZ})} {{cov(\Delta}_{jXY,XZ}) \atop{var(\Delta}_{jXZ})} \right)$ | (4) |
| --- | --- | --- |
|  |  |  |

All the terms of the covariance matrix ${\boldsymbol{W}_{\boldsymbol{jg}}}^{\boldsymbol{-1}}$ can be obtained by bootstrapping.

Given the expected heterogeneity, we will use a random effect model, all the equations used in the standard meta-analysis approach hold, but assume a vectorial form instead than a scalar one. For instance, for the group *S_j_* we have:

|  | $\boldsymbol{\Delta}_{g}=\left( \Delta_{jXY}, \Delta_{jXZ} \right)^{T}=\frac{\sum_{j} \mathbf{W}_{\mathrm{jg}}\Delta_{\mathrm{jg}}}{\sum_{j} \mathbf{W}_{\mathrm{jg}}}$, | (5) |
| --- | --- | --- |
|  |  |  |

Which has the covariance matrix

|  | ${W_{jg}}^{-1}=\left( \sum_{j} W_{jg} \right)^{-1}$. | (6) |
| --- | --- | --- |
|  |  |  |

##### **Stage II. Regression to consistent estimation**

We will follow the approach explained and implemented in [26].

# MSC: HETEROGENEITY

We have already defined the heterogeneity for meta-analysis of direct comparisons. A similar definition can be used for the heterogeneity of network meta-analysis, adapting a definition used for multi-arm trials to multiple score comparison. Since we have singleton groups in our MSC data (group 6 in our database), it is recommended in our case to use pooled estimate of the *τ^2^* (*τ^2^_pooled_*), [26] defined as follows:

$${\tau^{2}}_{pooled}=\frac{\sum_{g} Q_{g}-\sum_{g} df_{g}}{\sum_{g} C_{g}}$$

i.e. a multivariate version of the pooled estimate for the heterogeneity variance.

## Tau pooled vs tau group

An alternative (not recommended in our case) is to consider not a pooled version of *τ^2^*, but a *τ^2^* for each group (*τ^2^_g_*), defined as follows:

$$Q_{g}=\sum_{j} {(\boldsymbol{d}\boldsymbol{̂}}_{jg}-{\boldsymbol{d}\boldsymbol{̂}}_{g})W_{g}{(\boldsymbol{d}\boldsymbol{̂}}_{jg}-{\boldsymbol{d}\boldsymbol{̂}}_{g})$$

$$df=\left( T_{g}-1 \right)\left( N_{g}-1 \right)$$

$$C_{g}=tr\left\{ \sum_{j} W_{jg}-\sum_{j} W_{jg}^{2}\left( \sum_{j} W_{jg} \right)^{-1} \right\}$$

$${\taû}_{g}^{2}=max\{0,\frac{Q_{g}-{df}_{g}}{C_{g}}\}$$

Where

In the previous formulas the subscript *j* represents a generic cohort and the subscript *g* represents a generic group. Then, ***W****_jg_* is the inverse of the covariance matrix related to the performance of the scores in the cohort *j* in the group *g*. *T_g_* is the number of scores that can be evaluated in the group *g*, *N_g_* is the number of cohorts in the group *g*. Finally, **d̂_g_** is the estimate of the performance vector in the group *g* and **d̂_jg_** is the estimate of the performance vector in the cohort *j* of the group *g*.

| **Heterogeneity parameters in the MSC meta-analysis** | | | | | |
| --- | --- | --- | --- | --- | --- |
| **Group** | **Q_g_** | **df_g_** | **C_g_** | **τ_g_^2^** | **τ_g_** |
| 1 | 3 | 3 | 4667 | 0.000 | 0.00000 |
| 2 | 16 | 12 | 19105 | 0.014 | 0.00019 |
| 3 | 11 | 8 | 4699 | 0.025 | 0.00062 |
| 4 | 0 | 0 | 0 | 0.000 | 0.00000 |
| 5 | 48 | 30 | 148807 | 0.011 | 0.00012 |
| 6 | 35 | 24 | 57077 | 0.014 | 0.00018 |

We notice that the values for the singleton group 5 are all 0. This is the reason that induces us to consider more reliable the *τ^2^* pooled approach compared to the *τ^2^_g_* approach.

We show below a visual comparison between the estimates in the 2 cases:

- **τ_pooled_**
- **τ_g_**

**τ_pooled_**


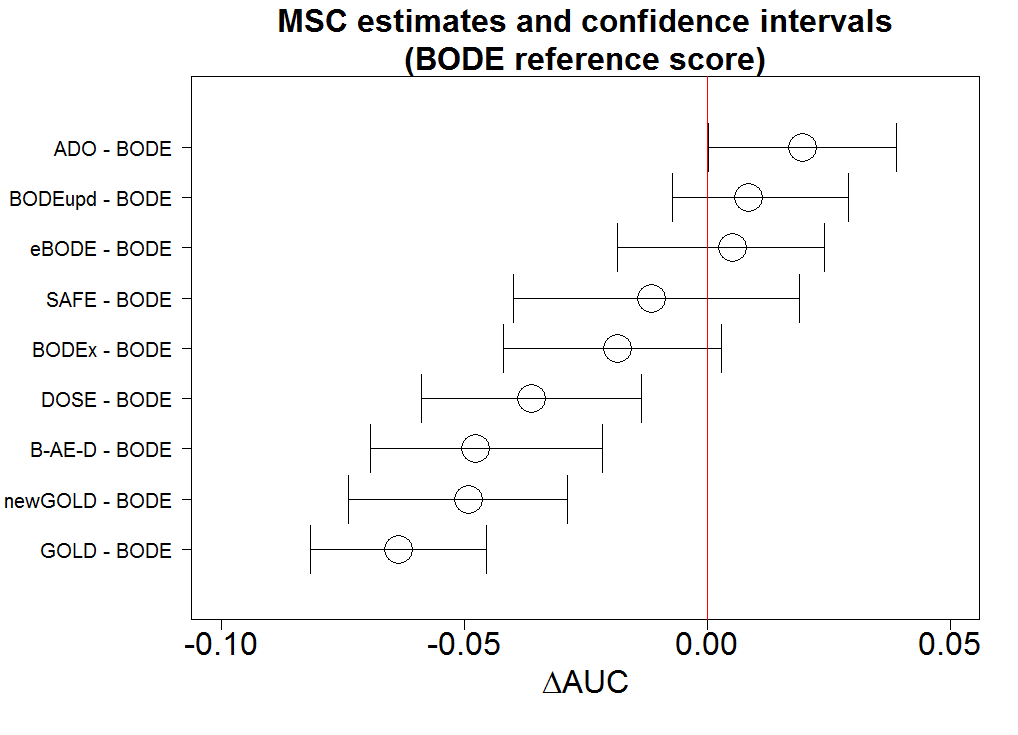


**τ_g_**


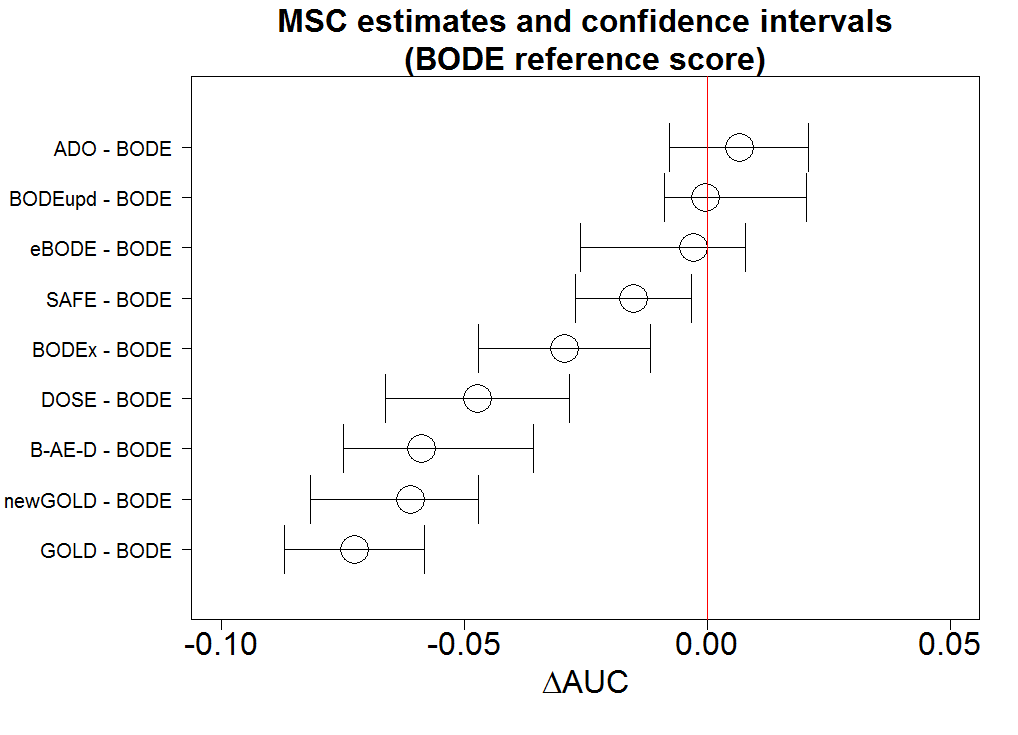


There are no differences in the ranking. Some differences in the CIs are to notice: the authors feel more comfortable with a much bigger CI for, say, the SAFE score as in the case of τ_pooled._ Indeed, we expect a MSC CI to be bigger for a score that is present in only 1 group (SAFE is only in group 4 that is constituted by only one cohort). Thus, the authors are convinced that the τ_pooled is the best_ approach for their case.

# MSC: TRANSITIVITY

For a brief introduction concerning the concept of transitivity (or similarity) please look at the section “transitivity (or similarity)” in the protocol above. For a more detailed discussion we recommend the suited literature [45–48].

A requirement for the application of a network meta-analytic technique is “transitivity”, i.e., in the case of treatment effect comparison, “similarity” in the distributions of “effect modifiers”[49] and in study-level characteristics (such as inclusion and exclusion criteria, details on subject recruitment, or study design choices).[50] In MSC we do not refer to similarity in the distributions of “effect modifiers” but to “spectrum effect”[51, 52] or “case mix”,[50, 53, 54] defined as the distribution of predictor values, other relevant participant or setting characteristics (such as therapy in stable conditions) and the outcome incidence.[53] Indeed, it is well established in prediction model literature that as the case-mix heterogeneity increases, individuals have a larger variety of patient characteristics, and the model tends to discriminate better.[55, 56]

In practice, we used the definition number 4 of transitivity from [48].

Thus, we evaluated by meta-regression analysis[57] the distribution of the variables that could generate case mix variation (like median and variability of age,[53] range and variance of obstruction severity (i.e., FEV1% pred.), exercise capacity, size, mortality rate). Then, we used analysis of variance (ANOVA) to see whether the distribution of the identified variables was imbalanced in the groups and could consequently generate imbalance in the performance group by group.

We tested if case-mix heterogeneity of the following variables can affect the discriminative power of the models, namely the AUC):

- Range of obstruction severity (i.e., FEV1% pred.)
- Variance of obstruction severity (i.e., FEV1% pred.)
- Mortality percentage
- Exercise capacity range
- Median of age
- Variability of age[53]
- Size (i.e., number of events)

In case of a variables directly affecting the performance, we used analysis of variance (ANOVA) to see whether the distribution of the identified variables was imbalanced in the groups and could consequently generate imbalance in the performance group by group. In case of homogenous groups, we cannot reject the null hypothesis of transitivity.

For the following 2 variables the meta-regression analysis informed us that they were not generating case-mix when looking at the differences in AUC.

- Variability of age[53]
- Size (i.e., number of events)

Instead, the following 5 variables were significantly affecting the differences in AUC. Then, they generate case-mix.

- Range of obstruction severity (i.e., FEV1% pred.)
- Variance of obstruction severity (i.e., FEV1% pred.)
- Mortality percentage
- Exercise capacity range
- Median of age

Thus, for these variables, we had to check if they are balanced across groups to verify that transitivity holds. The ANOVA tests ensured the balance of these variables in our groups of the MSC meta-analysis.

In conclusion, we can assume that the transitivity assumption holds for our network.

## AUC vs FEV1 %pred. range


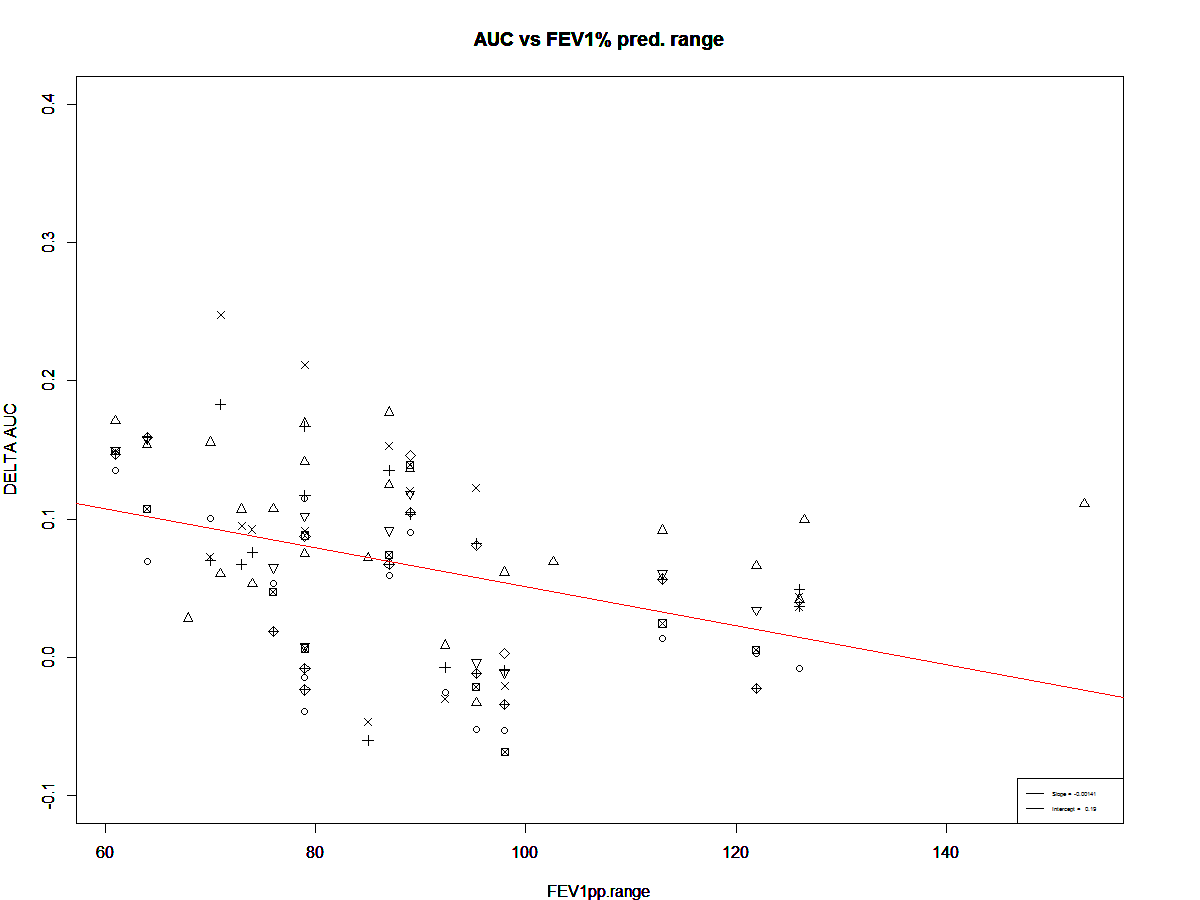


Interesting to see that the slope has a negative coefficient for the ΔAUC (while we were expecting a positive coefficient for the meta-regression of AUC vs FEV1pp range)

> summary(regression.DELTA.AUC.vs.FEV1pp.range)

Call:

lm(formula = matrix_model_cohort.DELTA.AUC.not.NA.DELTA.AUC ~

matrix.FEV1pp.range.not.NA.DELTA.AUC)

Residuals:

Min 1Q Median 3Q Max

-0.13266 -0.04717 0.00697 0.04323 0.15552

Coefficients:

Estimate Std. Error t value

(Intercept) 0.1923504 0.0302376 6.361

matrix.FEV1pp.range.not.NA.DELTA.AUC -0.0014112 0.0003343 -4.221

Pr(>|t|)

(Intercept) 0.0000000059 ***

matrix.FEV1pp.range.not.NA.DELTA.AUC 0.0000532750 ***

---

Signif. codes: 0 ‘***’ 0.001 ‘**’ 0.01 ‘*’ 0.05 ‘.’ 0.1 ‘ ’ 1

Residual standard error: 0.06326 on 101 degrees of freedom

Multiple R-squared: 0.1499, Adjusted R-squared: 0.1415

F-statistic: 17.82 on 1 and 101 DF, p-value: **0.00005328**


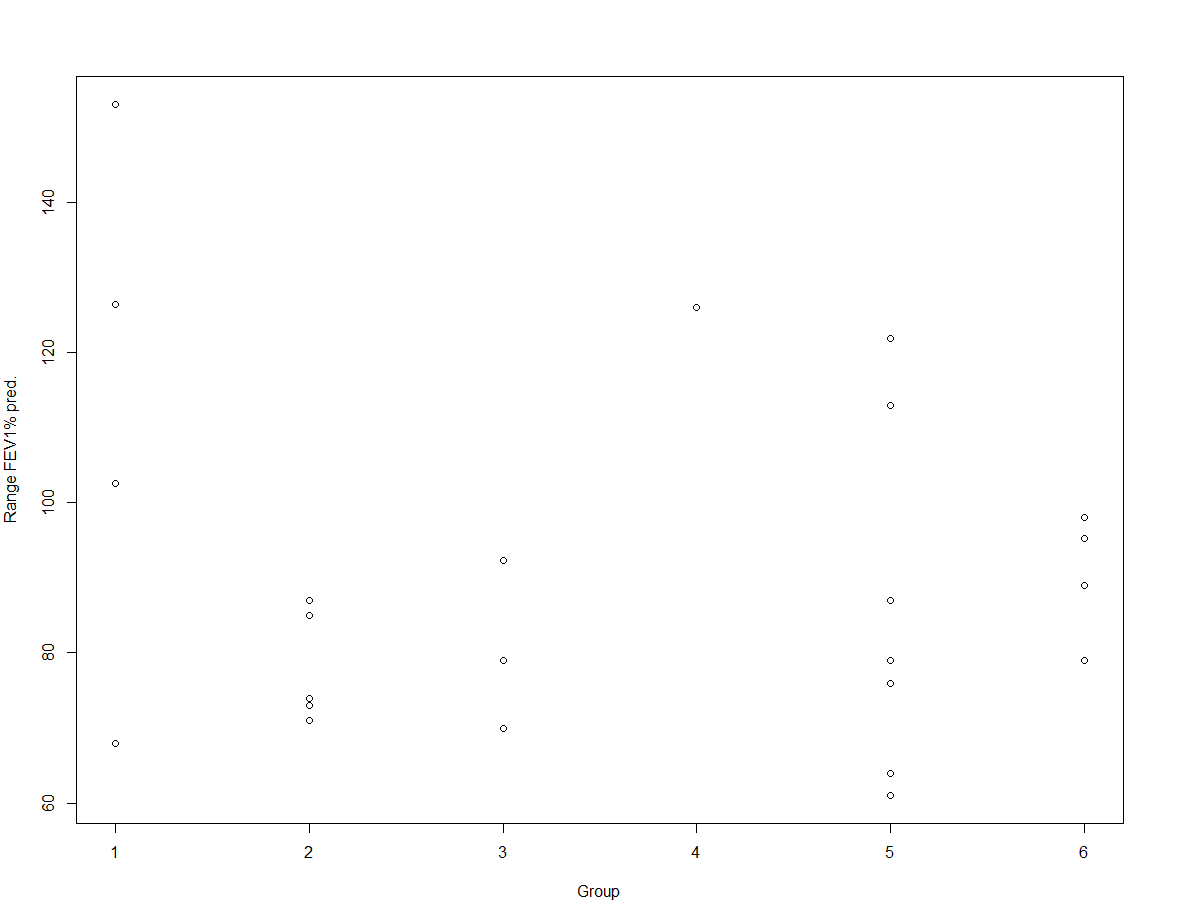


> anova(lm(FEV1pp.range ~ group.cohort))

Analysis of Variance Table

Response: FEV1pp.range

Df Sum Sq Mean Sq F value Pr(>F)

group.cohort 1 261.9 261.92 0.4777 **0.4967**

Residuals 22 12061.6 548.25

## AUC vs FEV1% pred. variance


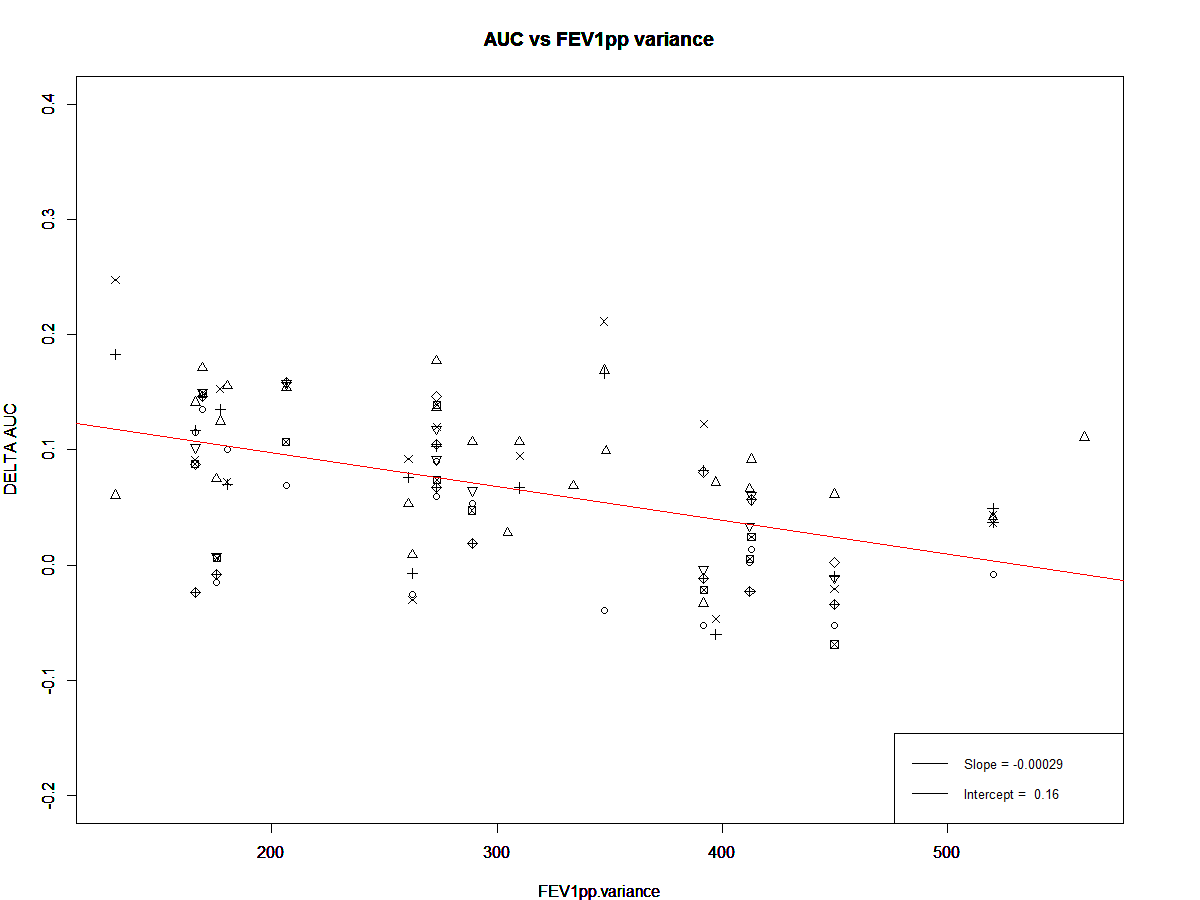


> summary(regression.DELTA.AUC.vs.FEV1pp.variance)

Call:

lm(formula = matrix_model_cohort.DELTA.AUC.not.NA.DELTA.AUC ~

matrix.FEV1pp.variance.not.NA.DELTA.AUC)

Residuals:

Min 1Q Median 3Q Max

-0.130903 -0.034511 0.002037 0.040787 0.157578

Coefficients:

Estimate Std. Error t value Pr(>|t|)

(Intercept) 0.15606493 0.01707820 9.138 7.09e-15 ***

matrix.FEV1pp.variance.not.NA.DELTA.AUC -0.00029364 0.00005309 -5.531 2.51e-07 ***

---

Signif. codes: 0 ‘***’ 0.001 ‘**’ 0.01 ‘*’ 0.05 ‘.’ 0.1 ‘ ’ 1

Residual standard error: 0.06011 on 101 degrees of freedom

Multiple R-squared: 0.2325, Adjusted R-squared: 0.2249

F-statistic: 30.6 on 1 and 101 DF, p-value: **0.0000002507**


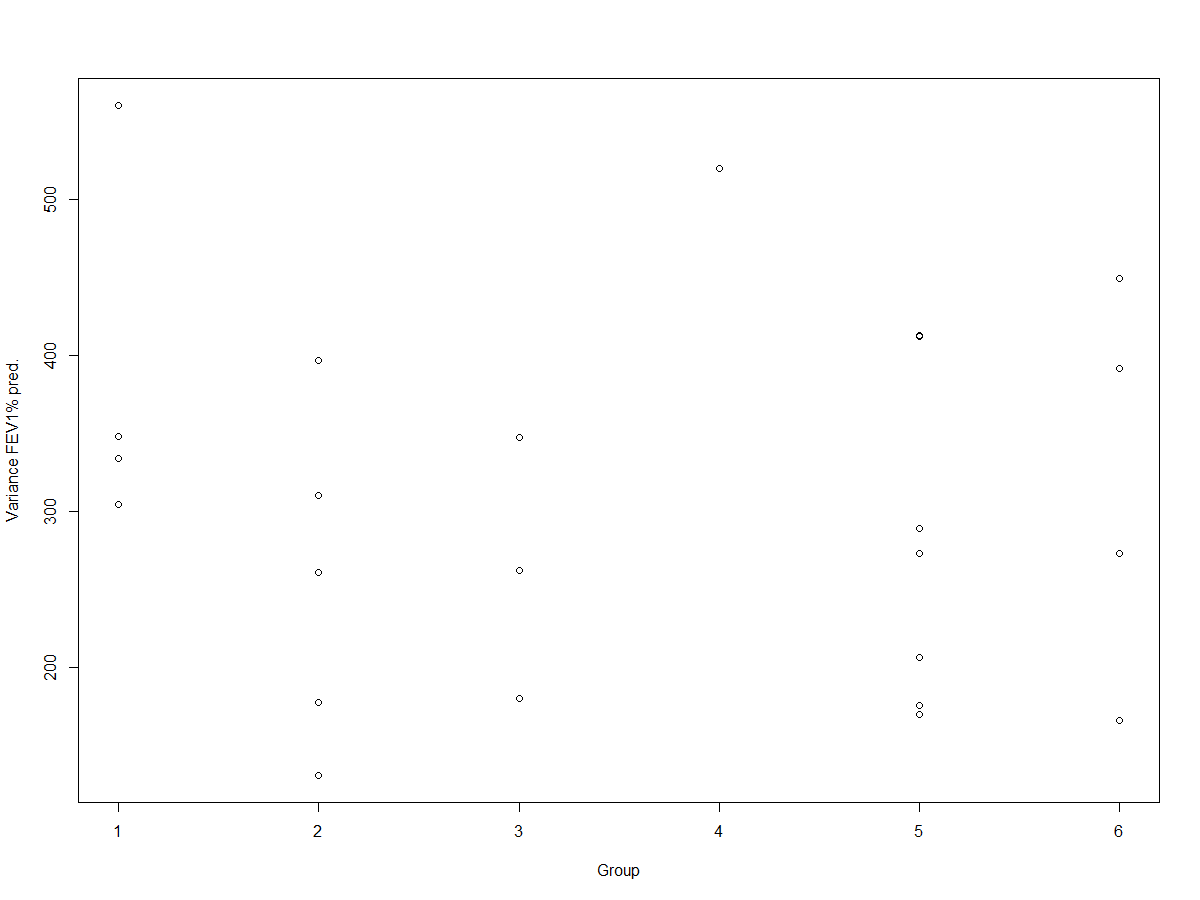


> anova(lm(FEV1pp.variance ~ group.cohort))

Analysis of Variance Table

Response: FEV1pp.variance

Df Sum Sq Mean Sq F value Pr(>F)

group.cohort 1 2276 2275.8 0.1635 **0.6898**

Residuals 22 306162 13916.5

## AUC vs mortality percentage


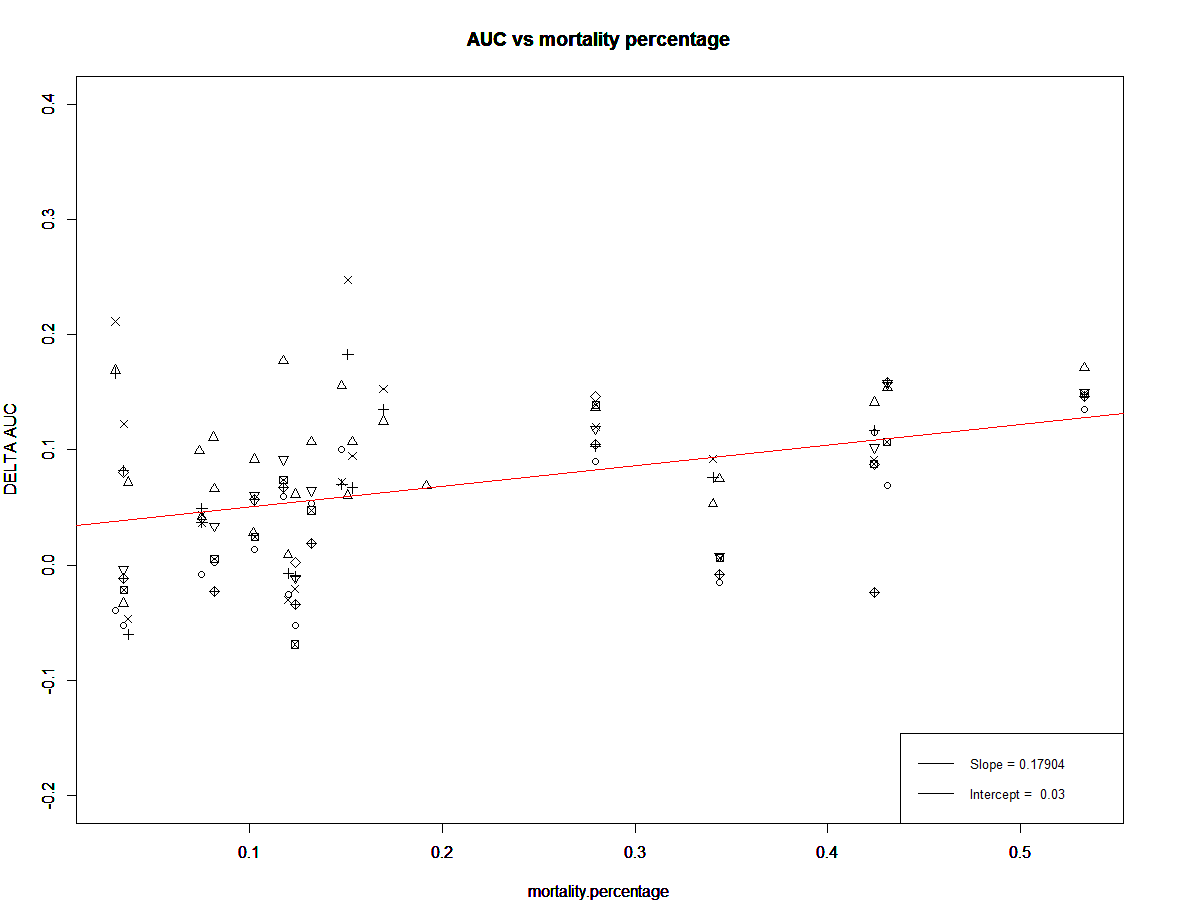


> summary(regression.DELTA.AUC.vs.mortality.percentage)

Call:

lm(formula = matrix_model_cohort.DELTA.AUC.not.NA.DELTA.AUC ~

matrix.mortality.percentage.not.NA.DELTA.AUC)

Residuals:

Min 1Q Median 3Q Max

-0.131970 -0.043406 0.005876 0.039659 0.188264

Coefficients:

Estimate Std. Error t value Pr(>|t|)

(Intercept) 0.03239 0.01035 3.129 0.0023 **

matrix.mortality.percentage.not.NA.DELTA.AUC 0.17904 0.04222 4.241 0.0000494 ***

---

Signif. codes: 0 ‘***’ 0.001 ‘**’ 0.01 ‘*’ 0.05 ‘.’ 0.1 ‘ ’ 1

Residual standard error: 0.06321 on 101 degrees of freedom

Multiple R-squared: 0.1512, Adjusted R-squared: 0.1428

F-statistic: 17.99 on 1 and 101 DF, p-value: **0.00004936**


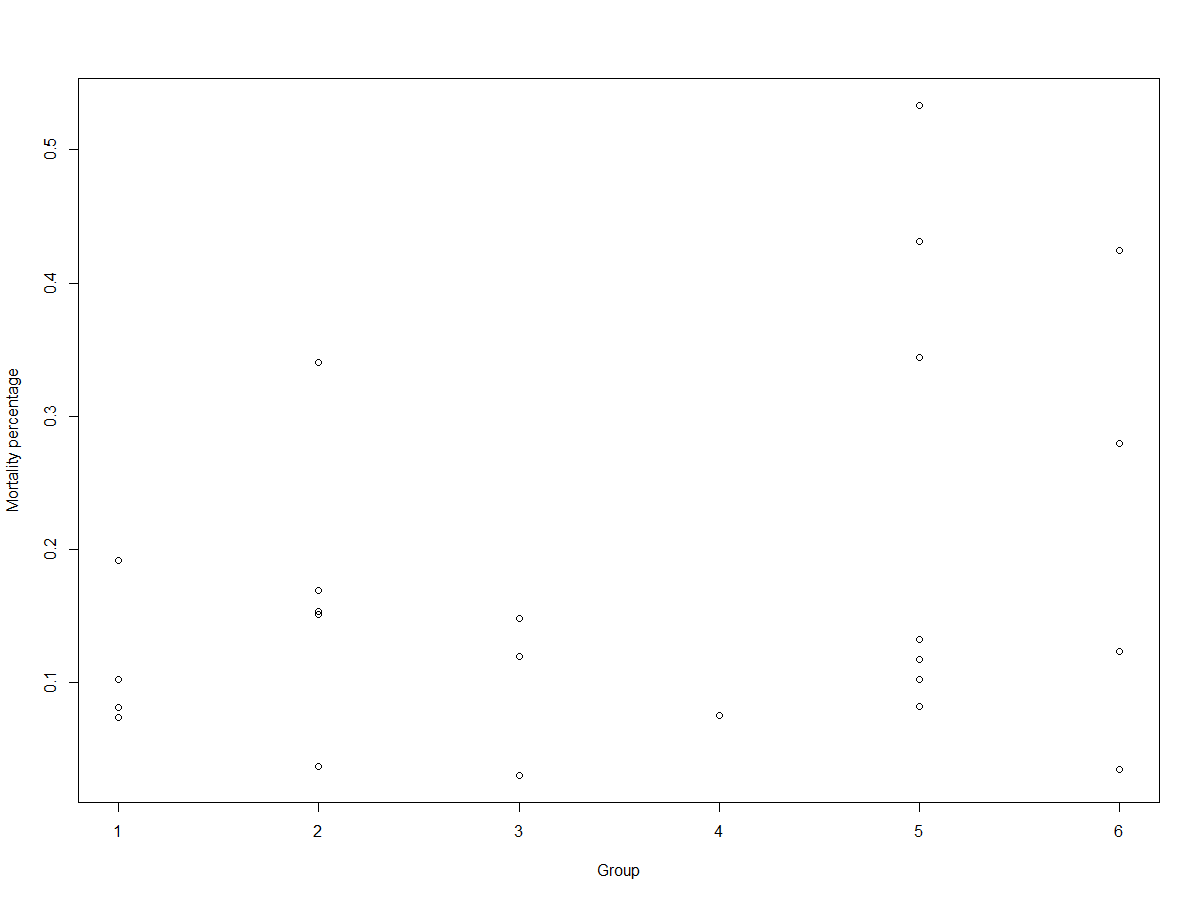


> anova(lm(FEV1pp.variance ~ group.cohort))

Analysis of Variance Table

Response: FEV1pp.variance

Df Sum Sq Mean Sq F value Pr(>F)

group.cohort 1 2276 2275.8 0.1635 **0.6898**

Residuals 22 306162 13916.5

## AUC vs exercise capacity range


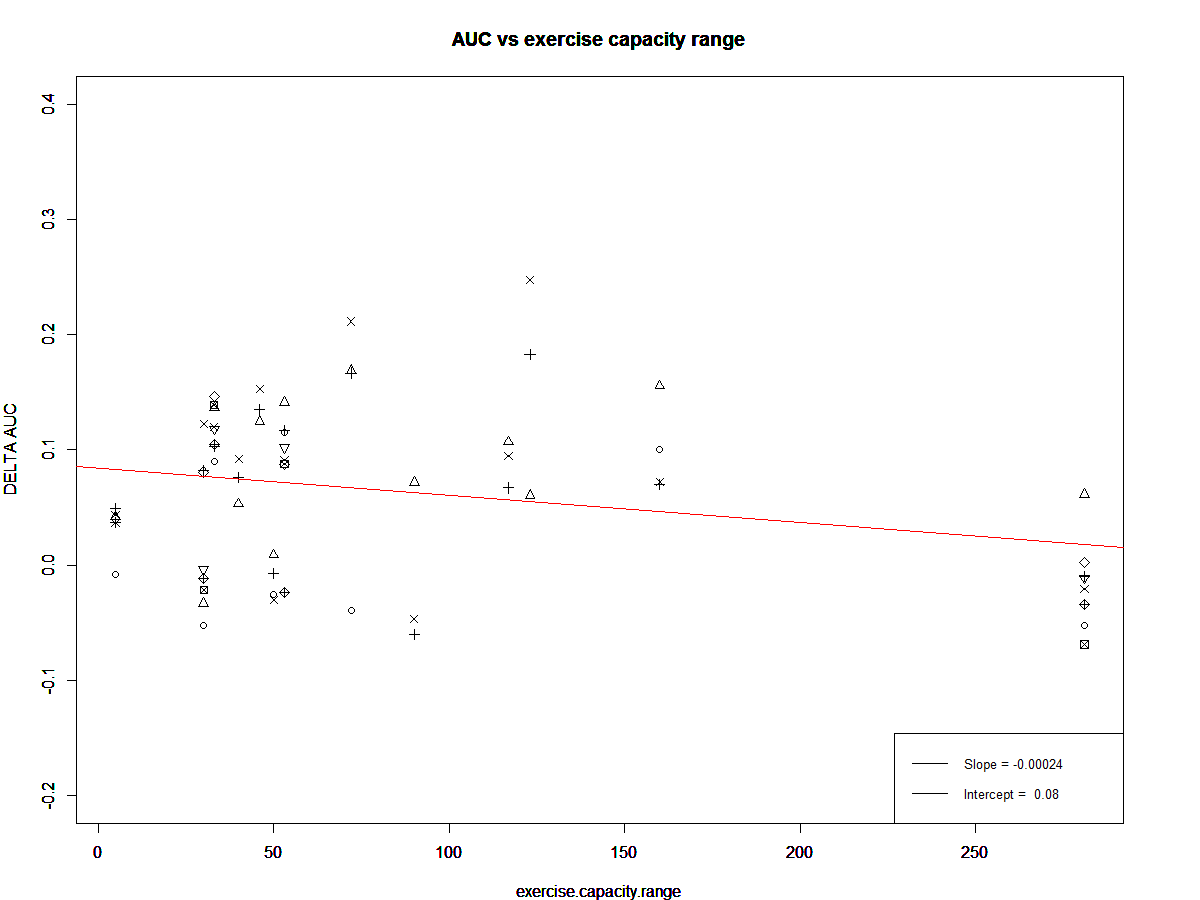


> summary(regression.DELTA.AUC.vs.exercise.capacity.range)

Call:

lm(formula = matrix_model_cohort.DELTA.AUC.not.NA.DELTA.AUC ~

matrix.exercise.capacity.range.not.NA.DELTA.AUC)

Residuals:

Min 1Q Median 3Q Max

-0.12926 -0.05526 0.01200 0.04527 0.19236

Coefficients:

Estimate Std. Error

(Intercept) 0.0843041 0.0131377

matrix.exercise.capacity.range.not.NA.DELTA.AUC -0.0002356 0.0001093

t value Pr(>|t|)

(Intercept) 6.417 0.0000000217

matrix.exercise.capacity.range.not.NA.DELTA.AUC -2.156 0.0349

(Intercept) ***

matrix.exercise.capacity.range.not.NA.DELTA.AUC *

---

Signif. codes: 0 ‘***’ 0.001 ‘**’ 0.01 ‘*’ 0.05 ‘.’ 0.1 ‘ ’ 1

Residual standard error: 0.07243 on 62 degrees of freedom

(39 observations deleted due to missingness)

Multiple R-squared: 0.06977, Adjusted R-squared: 0.05476

F-statistic: 4.65 on 1 and 62 DF, p-value: 0.03494


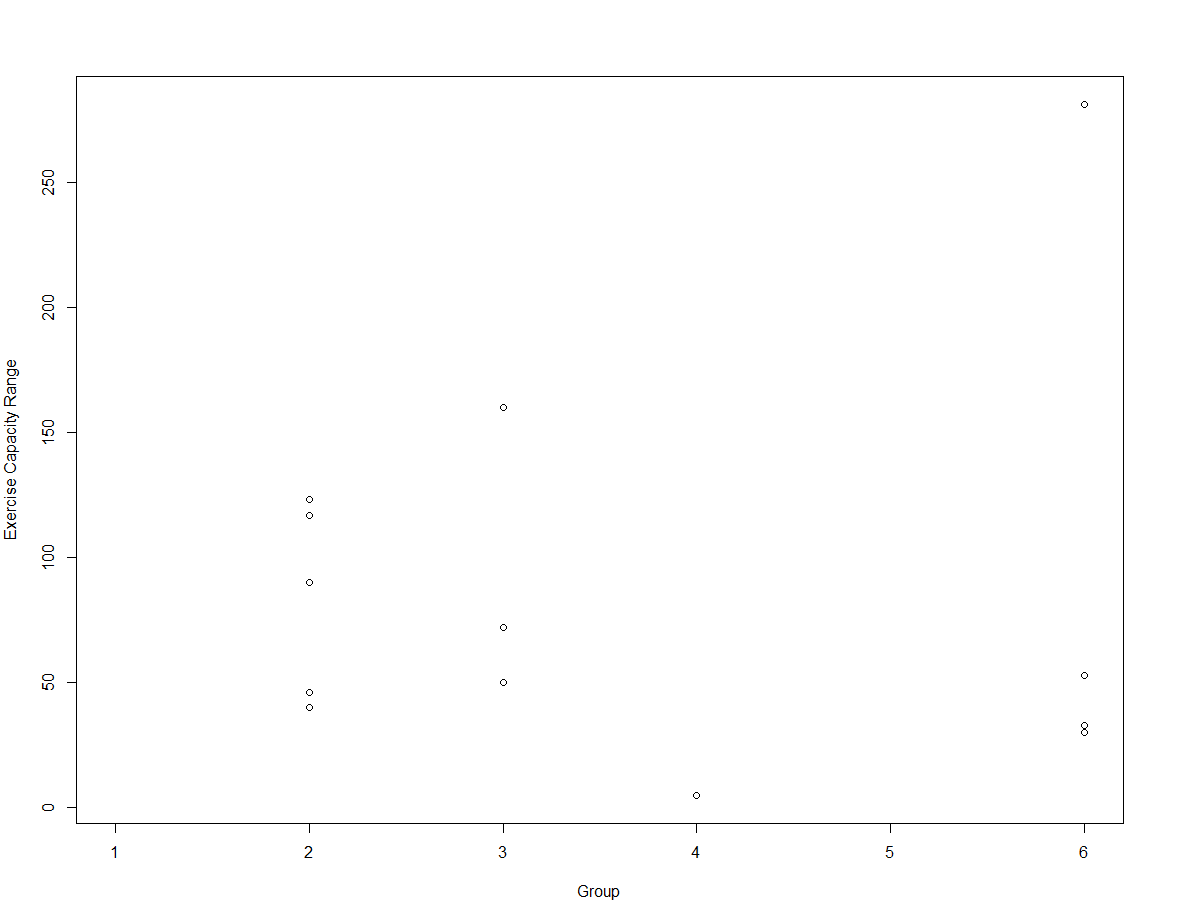


> anova(lm(exercise.capacity.range ~ group.cohort))

Analysis of Variance Table

Response: exercise.capacity.range

Df Sum Sq Mean Sq F value Pr(>F)

group.cohort 1 287 287.4 0.0491 0.8287

Residuals 11 64349 5849.9

## AUC vs median age


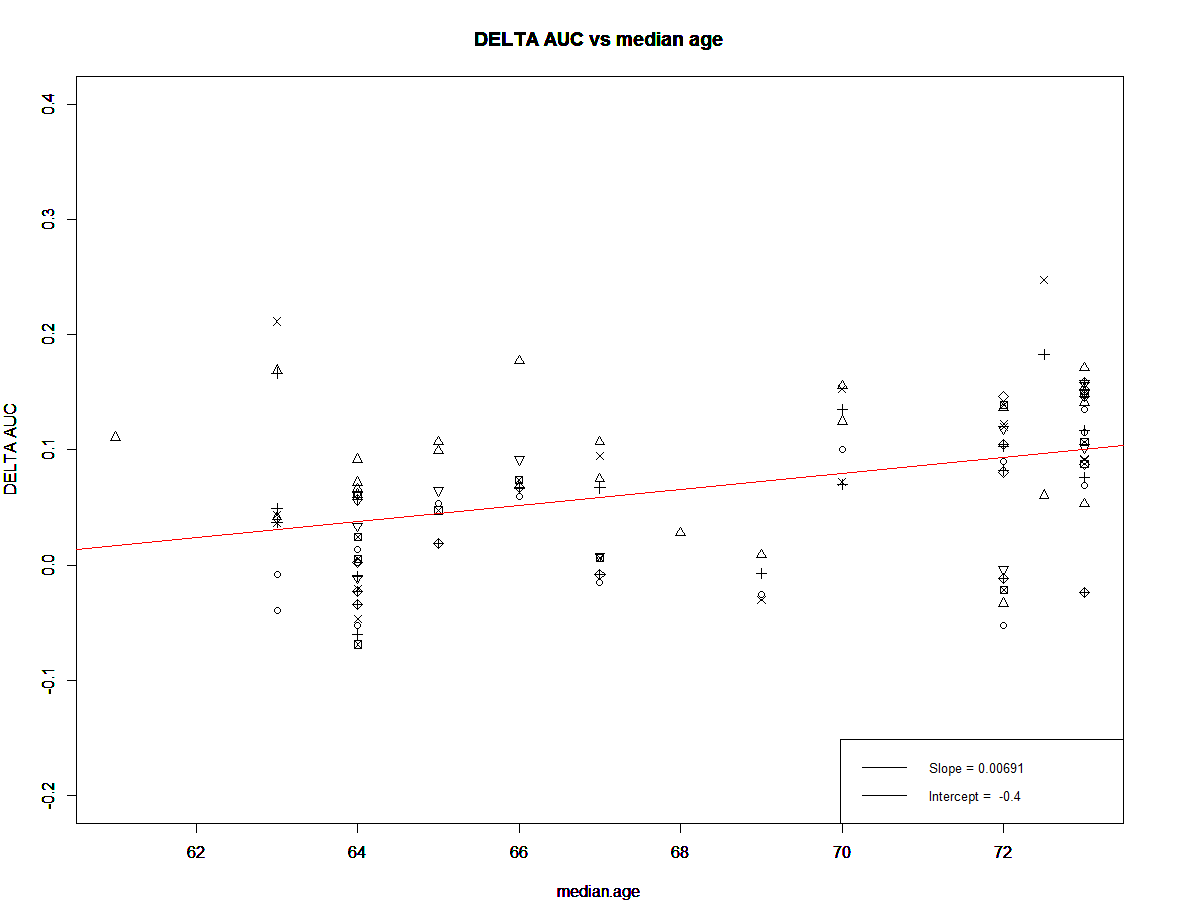


> summary(regression.DELTA.AUC.vs.median.age)

Call:

lm(formula = matrix_model_cohort.DELTA.AUC.not.NA.AUC ~ matrix.median.age.not.NA.DELTA.AUC)

Residuals:

Min 1Q Median 3Q Max

-0.14534 -0.03859 0.00828 0.04039 0.18044

Coefficients:

Estimate Std. Error

(Intercept) -0.404036 0.108947

matrix.median.age.not.NA.DELTA.AUC 0.006908 0.001594

t value Pr(>|t|)

(Intercept) -3.709 0.000341 ***

matrix.median.age.not.NA.DELTA.AUC 4.335 0.0000346 ***

---

Signif. codes: 0 ‘***’ 0.001 ‘**’ 0.01 ‘*’ 0.05 ‘.’ 0.1 ‘ ’ 1

Residual standard error: 0.063 on 101 degrees of freedom

Multiple R-squared: 0.1569, Adjusted R-squared: 0.1485

F-statistic: 18.79 on 1 and 101 DF, p-value: 0.00003455


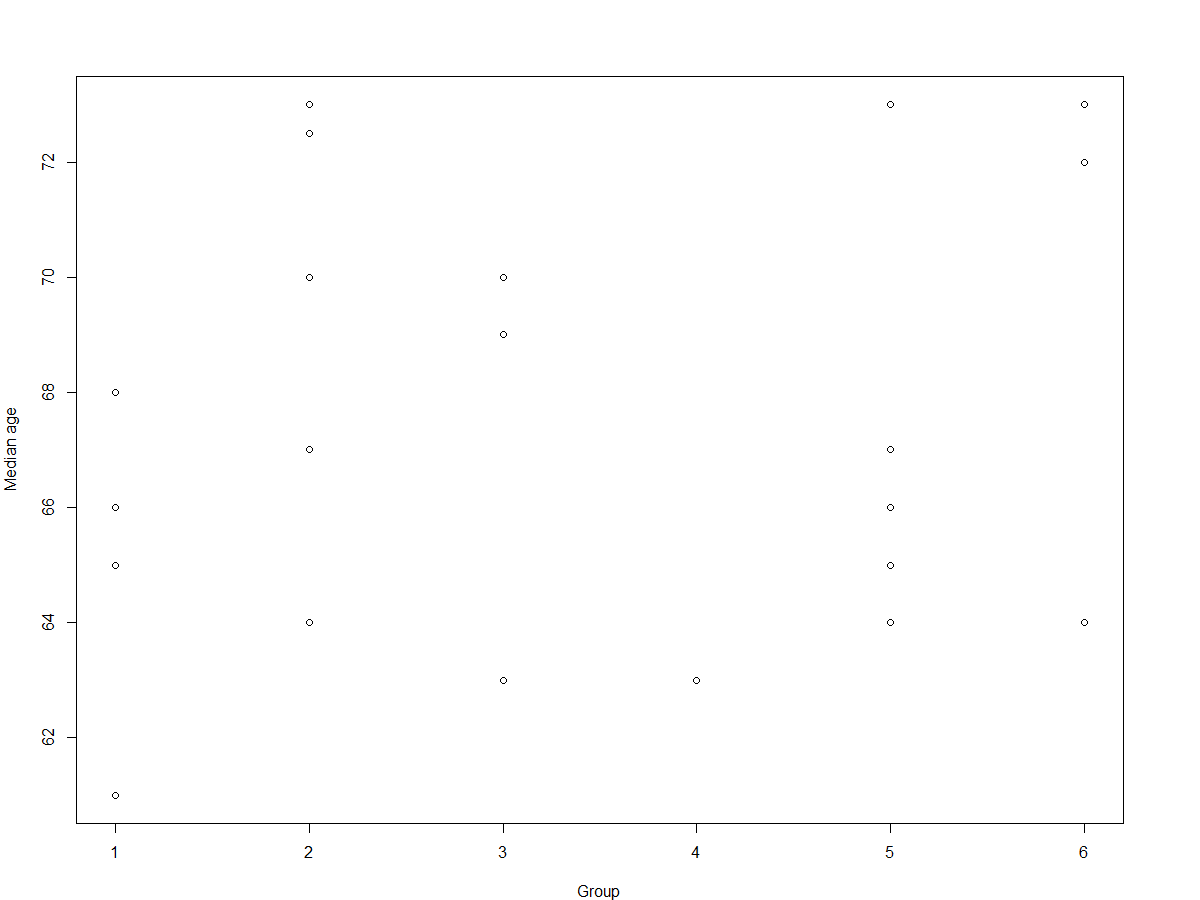


> anova(lm(median.age ~ group.cohort))

Analysis of Variance Table

Response: median.age

Df Sum Sq Mean Sq F value Pr(>F)

group.cohort 1 16.54 16.535 1.0912 0.3075

Residuals 22 333.37 15.153

## AUC vs variance age


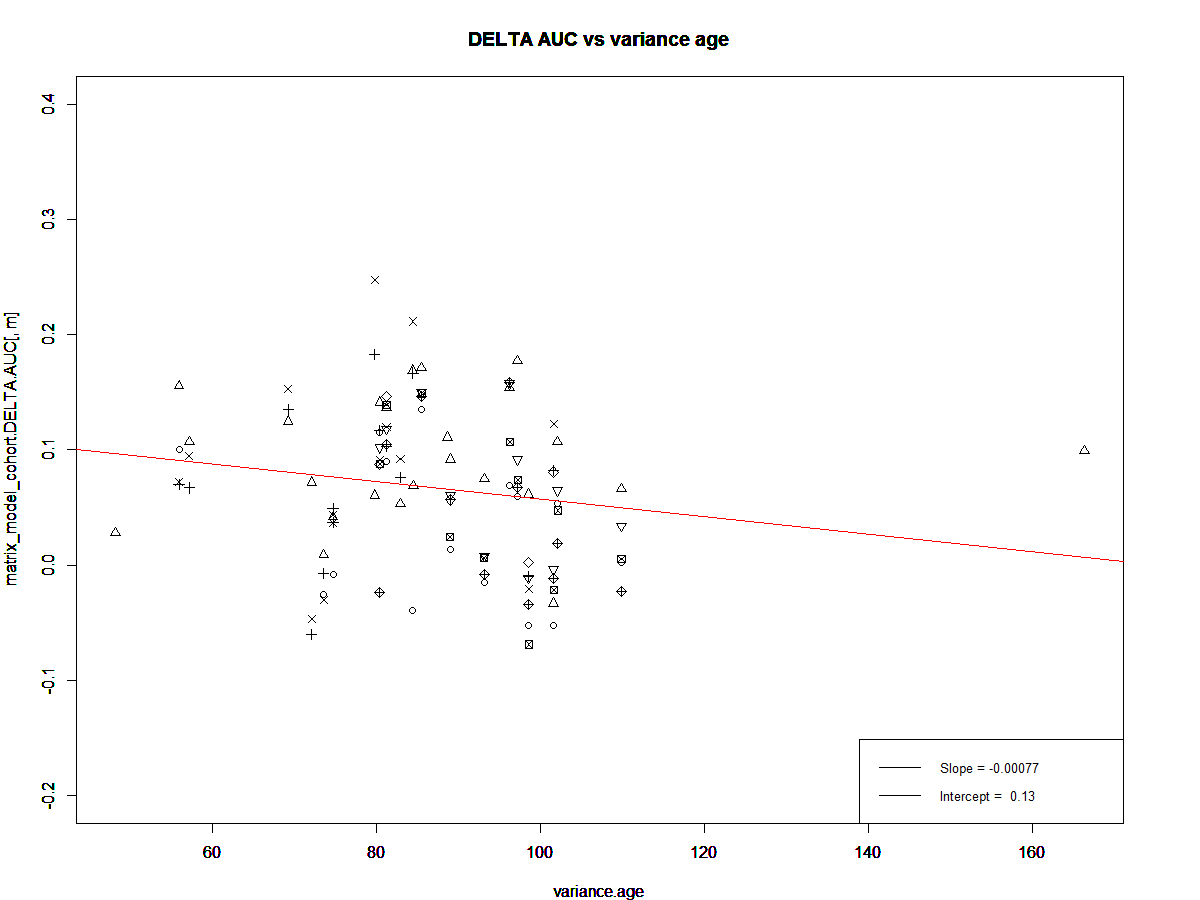


> summary(regression.DELTA.AUC.vs.variance.age)

Call:

lm(formula = matrix_model_cohort.DELTA.AUC.not.NA.DELTA.AUC ~

matrix.variance.age.not.NA.DELTA.AUC)

Residuals:

Min 1Q Median 3Q Max

-0.139115 -0.055526 0.005361 0.046485 0.174704

Coefficients:

Estimate Std. Error

(Intercept) 0.1340339 0.0370021

matrix.variance.age.not.NA.DELTA.AUC -0.0007652 0.0004184

t value Pr(>|t|)

(Intercept) 3.622 0.000459 ***

matrix.variance.age.not.NA.DELTA.AUC -1.829 0.070362 .

---

Signif. codes: 0 ‘***’ 0.001 ‘**’ 0.01 ‘*’ 0.05 ‘.’ 0.1 ‘ ’ 1

Residual standard error: 0.0675 on 101 degrees of freedom

Multiple R-squared: 0.03206, Adjusted R-squared: 0.02247

F-statistic: 3.345 on 1 and 101 DF, p-value: **0.07036**

## AUC vs size cohort (#cases)


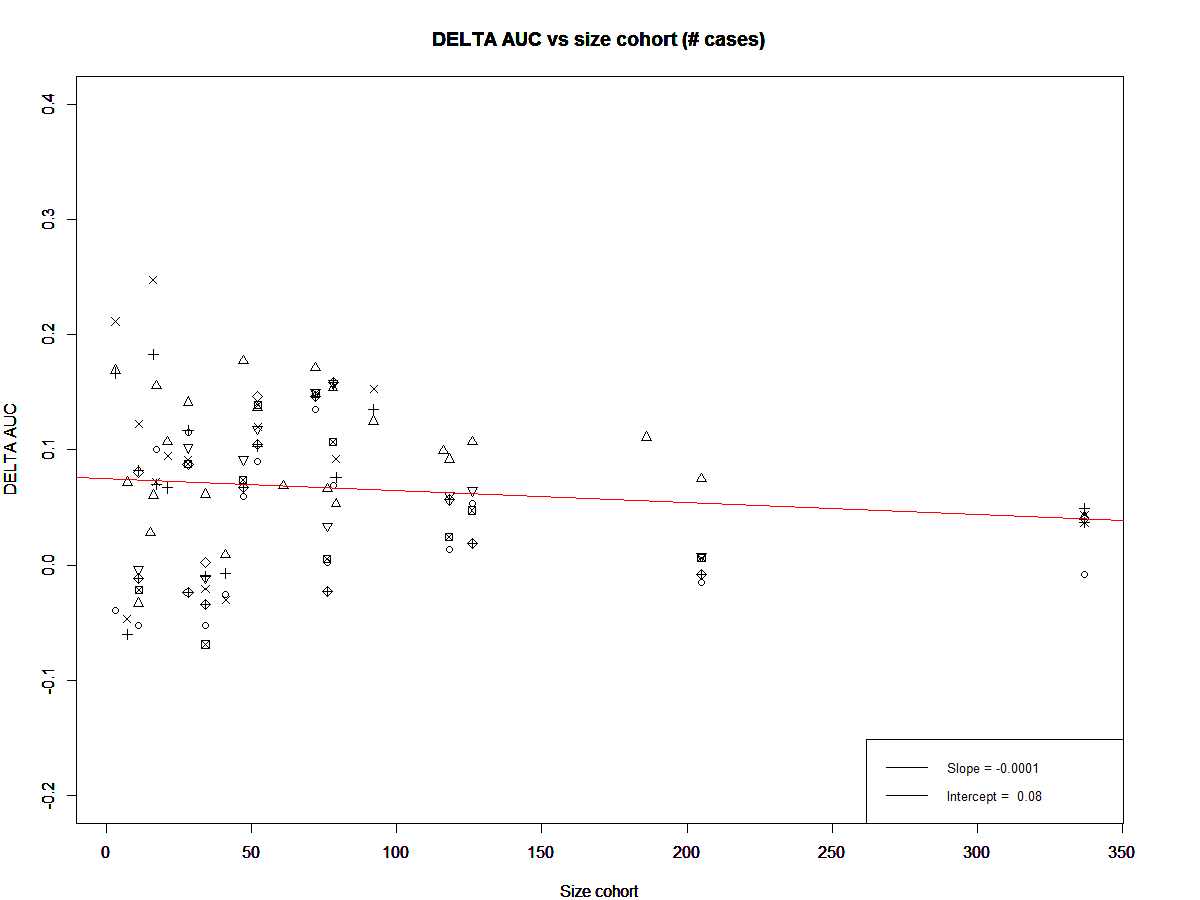


> summary(regression.DELTA.AUC.vs.size.cohort)

Call:

lm(formula = matrix_model_cohort.DELTA.AUC.not.NA.DELTA.AUC ~

matrix.size.cohort.not.NA.DELTA.AUC)

Residuals:

Min 1Q Median 3Q Max

-0.14034 -0.04871 0.00290 0.04680 0.17424

Coefficients:

Estimate Std. Error

(Intercept) 0.07510692 0.00926887

matrix.size.cohort.not.NA.DELTA.AUC -0.00010413 0.00008706

t value Pr(>|t|)

(Intercept) 8.103 1.29e-12 ***

matrix.size.cohort.not.NA.DELTA.AUC -1.196 0.234

---

Signif. codes: 0 ‘***’ 0.001 ‘**’ 0.01 ‘*’ 0.05 ‘.’ 0.1 ‘ ’ 1

Residual standard error: 0.06813 on 101 degrees of freedom

Multiple R-squared: 0.01397, Adjusted R-squared: 0.004205

F-statistic: 1.431 on 1 and 101 DF, p-value: **0.2345**

# INCOSISTENCY

Local and global inconsistency are evaluated by the following tests.[26]
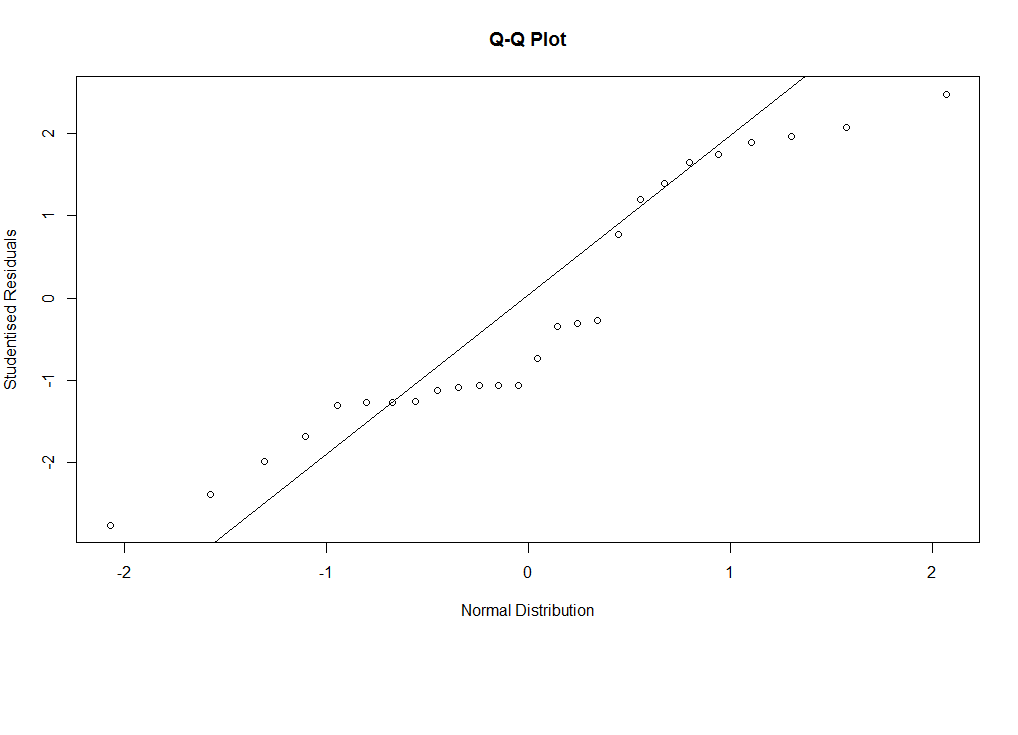


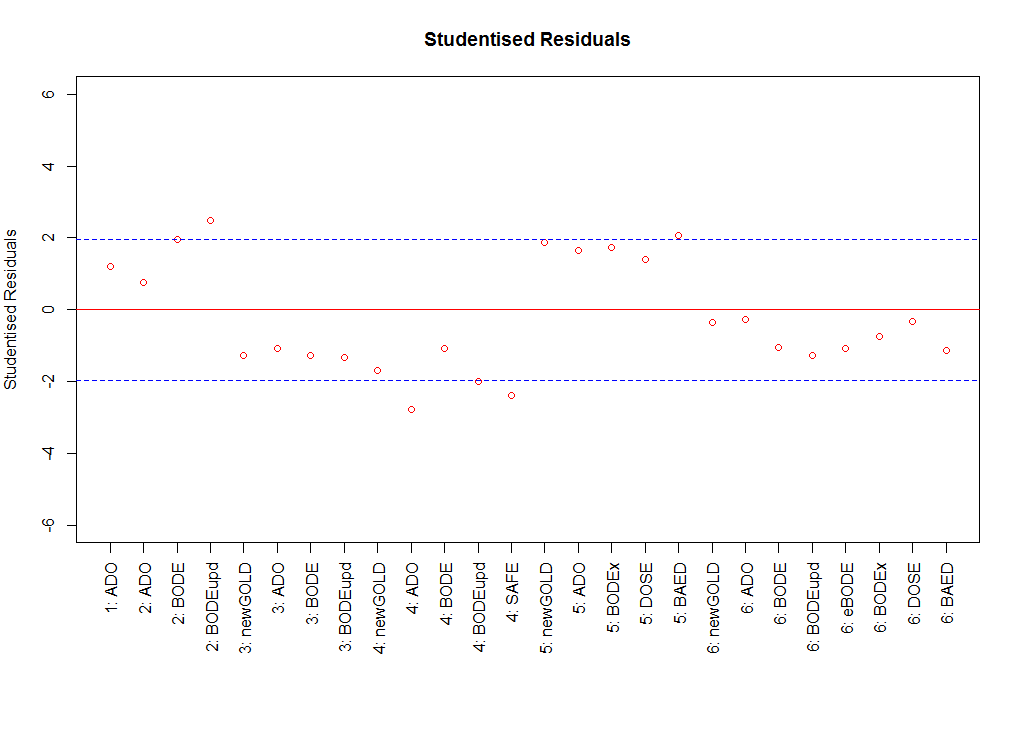


| Overall inconsistency parameters | |
| --- | --- |
| **Parameters** | **Value** |
| **α** | 0.05 |
| **K** | 9 |
| **df** | 16 |
| **Q** | 22.1 |
| **Χ^2^_df_** | 26.3 |
| **p-value** | 0.14 |
| 1-α = level of significance, K = # of scores; df = degrees of freedom (N-K+1, where N is the sum of all the scores used in the groups (28); Q = likelihood-ratio test statistic; Χ^2^_df_ = Χ^2^ statistic with df degrees of freedom | |

Our tests show a reasonably good local and global inconsistency.

# INDIRECT EVIDENCE

The following league table provides us with evidence of a reasonable consistency between direct and indirect evidence.

| **League table showing direct and indirect evidence** | | | | | | | | | | |
| --- | --- | --- | --- | --- | --- | --- | --- | --- | --- | --- |
| **Direct Indirect** | **GOLD** | **New**  **GOLD** | **ADO** | **BODE** | **BODE**  **upd** | **eBODE** | **BODEx** | **DOSE** | **SAFE** | **BAED** |
| **GOLD** | - | **0.022**  [-0.002, 0.047] | **0.090** [0.071, 0.109] | **0.067** [0.034, 0.099] | **0.075** [0.032, 0.117] | **0.074** [-0.009, 0.157] | **0.064** [0.033, 0.096] | **0.046** [0.012, 0.080] | **0.037** [0.015, 0.059] | **0.039** [0.003, 0.076] |
| **New GOLD** |  | - | **0.063** [0.050, 0.077] | **0.052** [0.037, 0.068] | **0.041** [0.008, 0.073] | **0.054** [0.015, 0.092] | **0.028** [0.017, 0.040] | **0.010** [-0.002, 0.023] | **0.045** [0.027, 0.063] | **0.007** [-0.018, 0.031] |
| **ADO** |  |  | - | **-0.007** [-0.031, 0.017] | **-0.004** [-0.036, 0.029] | **-0.018** [-0.079, 0.043] | **-0.045** [-0.062, -0.027] | **-0.064** [-0.084, -0.044] | **-0.005** [-0.028, 0.018] | **-0.068** [-0.090, -0.045] |
| **BODE** |  | **0.048** [0.016, 0.079] |  | - | **0.004** [-0.007, 0.015] | **0.010** [-0.017, 0.038] | **-0.005** [-0.039, 0.030] | **-0.030** [-0.094, 0.035] | **-0.012** [-0.025, 0.000] | **-0.041** [-0.099, 0.017] |
| **BODE upd** |  | **0.076** [0.041, 0.111] |  |  | - | **0.012** [-0.018, 0.041] | **-0.010** [-0.057, 0.037] | **-0.034** [-0.100, 0.032] | **-0.006** [-0.020, 0.008] | **-0.043** [-0.100, 0.014] |
| **eBODE** |  |  |  |  |  | - | **-0.023** [-0.045, -0.001] | **-0.040** [-0.087, 0.006] |  | **-0.053** [-0.098, -0.007] |
| **BODEx** |  |  |  | **-0.022** [-0.049, 0.004] | **-0.035** [-0.063, -0.007] |  | - | **-0.017** [-0.029, -0.005] |  | **-0.024** [-0.038, -0.009] |
| **DOSE** |  |  |  | **-0.041** [-0.066, -0.015] | **-0.052** [-0.079, -0.024] |  |  | - |  | **-0.005** [-0.025, 0.015] |
| **SAFE** |  |  |  |  |  | **-0.021** [-0.056, 0.014] | **0.010** [-0.022, 0.042] | **0.026** [-0.007, 0.059] | - |  |
| **BAED** |  |  |  | **-0.051** [-0.080, -0.022] | **-0.062** [-0.093, -0.032] |  |  |  | **-0.041** [-0.076, -0.005] | - |
| In the lower-left part of the matrix, the indirect comparisons are indicated; instead, in the upper-right part of the matrix the direct comparisons are indicated. Highlighted in red are the indirect comparisons and the direct comparisons, in the cases where the indirect comparison related to the same pair of scores is available as well. We note that in four cases (SAFE vs eBODE, BODEx, DOSE, BAED) the direct evidence is not available (cells colored in red), while the indirect evidence is available. | | | | | | | | | | |

# MULTIPLE IMPUTATION

We handle in the analysis for the main paper the missing data as follows: if a variable was missing for >30% of observations we discarded the specific variable for that particular specific cohort, since, in this case, we deemed the percentage of missing data too high not to be biased.

An alternative could be to still delete a variable in each cohort where it is missing >30% of observations and, in addition, to impute the missing data (related to that variable) by multiple imputation with chained equations (the analysis of the patterns of missingness allow us to consider the missing data missing completely at random apart from the dependence on the cohort).[7, 58, 59]). We imputed the missing data (related to that variable) by multiple imputation with chained equations[7, 58–61].

It could be argued that the choice of 30% as threshold to decide if to discard the variable in a particular cohort or to impute it, is partly arbitrary. In part for sure it is, but it is based on same general consideration concerning missing data and on our particular analysis.[7]

The pooled results for 10 imputed database follow.

**# Imputations = 0**


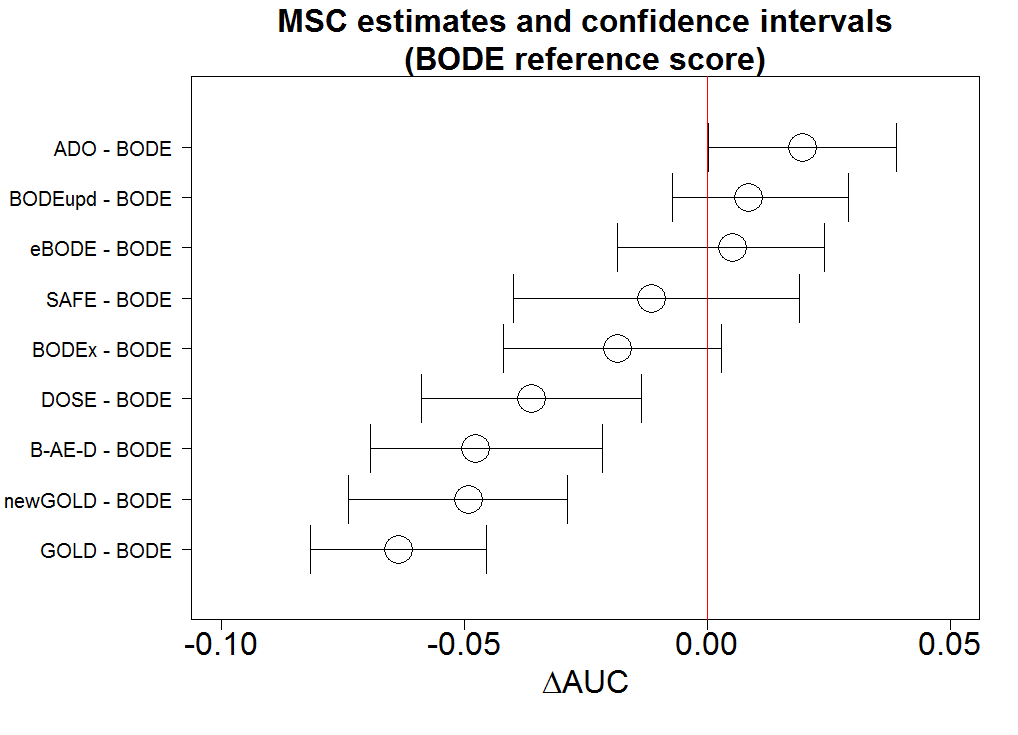


**# Imputations = 10**


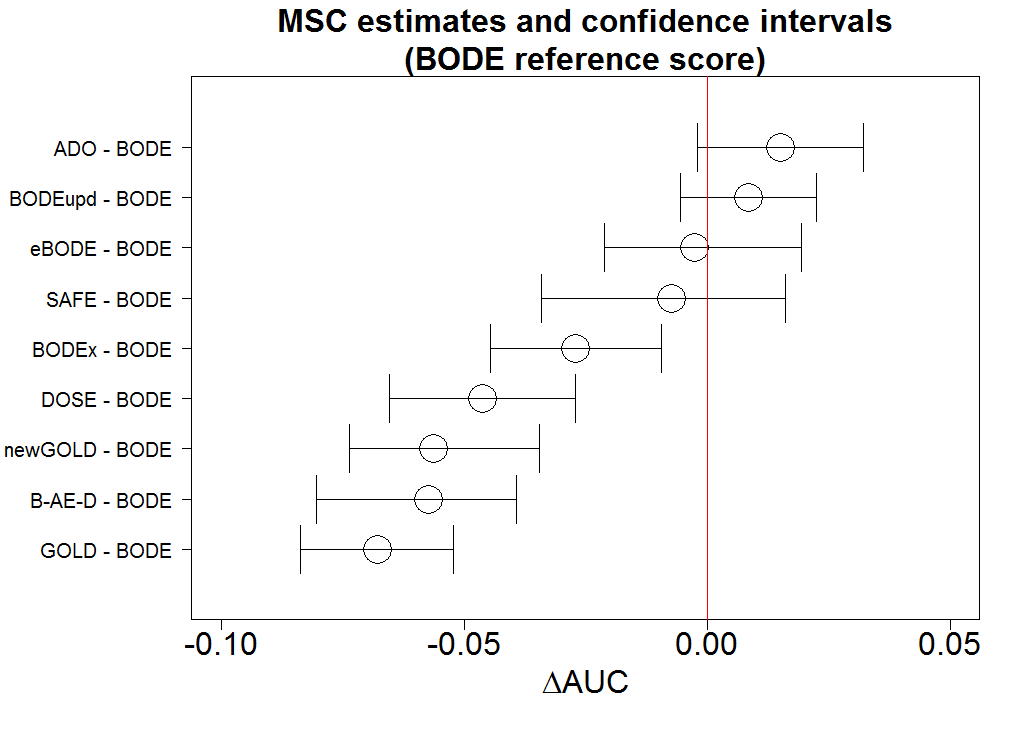


We note that there are no big differences between the analysis without and with imputation (10 imputations).

# SUCRA p-score

*As reported in the main paper, in order to provide a ranking of the scores, we used a frequentist version of the SUCRA score (a simple numerical summary to supplement the graphical display of cumulative ranking in model performance)*^14,15^*, i.e. the “p-score”.*^16^ *P̅_i_ represents the rank of score i within the given set of scores, where 1 means theoretically best and 0 means worst.*

| *Table 3\|P-Score (frequentist version of the SUCRA score)* | |
| --- | --- |
| ***Score*** | *P̅_i_* |
| ***ADO*** | *0.99* |
| ***BODE upd.*** | *0.80* |
| ***eBODE*** | *0.78* |
| ***BODE*** | *0.65* |
| ***SAFE*** | *0.55* |
| ***BODEx*** | *0.54* |
| ***DOSE*** | *0.32* |
| ***New GOLD*** | *0.21* |
| ***B-AE-D*** | *0.15* |
| ***GOLD*** | *0.02* |
| *P̅_i_ represents the rank of score i within the given range of scores, where 1 means theoretically best and 0 means worst.* | |

*The only needed check that we add in this supplementary material is that:*

|  | $\frac{1}{nScores}\sum_{i=1}^{nScores} \bar{P_{i}}=0.5$ | *(7)* |
| --- | --- | --- |
|  |  |  |

# CORRELATIONS AMONG SCORES

Plot showing the correlation among the performance among scores.

A for ADO, B for any of the BODE variants, G for GOLD, and  O for the others (SAFE, DOSE, BAED). So, AB below means ADO compared with any of the four BODE scores. B only means comparisons among the  BODE variants, and O alone means comparisons among the other scores. Median correlation was 0.67, but the IQR was relatively wide (0.54 -  0.78). One correlation was even negative (DOSE vs ADO in the largest  group). Sorting by the scores compared, lowest correlation was between  ADO and other scores, but even that was often above 0.5. Highest  correlation was (as we expected) between the BODE variants (the box on the extreme right), with median  correlation 0.83, with IQR 0.77 - 0.92.

It is to note (expected and intuitive) the strong correlation among some of the scores. Indeed, for instance, the biggest correlation is among the scores originated by the BODE score /(BODE, BODE update, eBODE, BODEx).

# SCORES

| Scoring rules of COPD multicomponent scores that we have validated in our study | | | | | | | | | | |
| --- | --- | --- | --- | --- | --- | --- | --- | --- | --- | --- |
| **Score**  **Predictor** | **GOLD**  [62] | **New**  **GOLD**  [62] | **BODE**  [63] | **BODE**  **upd.**  [64] | **ADO upd.**  [65] | **e-BODE**  [66] | **BODEx**  [66] | **DOSE**  [67] | **SAFE**  [68] | **B-AE-D**  **opt.**  [69] |
| **BMI** |  |  | 0 (>21)  1 (<=21) | 0 (>21)  1 (<=21) |  | 0 (>21)  1 (<=21) | 0 (>21)  1 (<=21) |  |  | 0 (>=21)  6 (18.5-21)  9 (<18.5) |
| **FEV1% pred.** | 0 (>=80)  1 (50-79)  2 (30-49)  3 (<30) | 0 (if FEV1pp>=50  and  <=1 exacerbations per year)  2 (otherwise) | 0 (>=65%)  1 (50-64%)  2 (36-49%)  3 (<=35) | 0 (>=65%)  1 (36-64%)  2 (<=35) | 0 (>=81%)  1 (65-60%)  2 (51-64%)  3 (35-50%)  4 (<=35%) | 0 (>=65%)  1 (50-64%)  2 (36-49%)  3 (<=35) | 0 (>=65%)  1 (50-64%)  2 (36-49%)  3 (<=35) | 0 (>=50%)  1 (31-49%)  2 (<=30) | 0 (>=80%)  1 (50-79%)  2 (36-49%)  3 (<=35) |  |
| **mMRC** |  | 0 (if mMRC >=2 and CAT >=10)  1 otherwise | 0 (0-1)  1 (2)  2 (3)  3 (4) | 0 (0-1)  1 (2)  2 (3)  3 (4) | 0 (0)  1 (1-2)  2 (3)  3 (4) | 0 (0-1)  1 (2)  2 (3)  3 (4) | 0 (0-1)  1 (2)  2 (3)  3 (4) | 0 (0-1)  1 (2)  2 (3)  3 (4) |  | 0 (0-2)  6 (3)  10 (4) |
| **6-MWT** |  |  | 0 (>=350)  1 (250-349)  2 (150-249)  3 (<=149) | 0 (>=350)  4 (250-349)  7 (150-249)  9 (<=149) |  | 0 (>=350)  1 (250-349)  2 (150-249)  3 (<=149) |  |  | 0 (>=400)  1 (300-399)  2 (200-299)  3 (<=199) |  |
| **Age** |  |  |  |  | 0 (40-49)  2 (50-59)  4 (60-69)  5 (70-79)  7 (>=80) |  |  |  |  |  |
| **Prev. exacerbations** |  | (See FEV1pp) |  |  |  | 0 (0)  1 (1-2)  2 (>2) | 0 (0)  1 (1-2)  2 (>2) | 0 (0-1)  1 (2-3)  2 (>3) |  | 0 (0)  3 (1)  7 (>=2) |
| **CAT** |  | (See mMRC) |  |  |  |  |  |  |  |  |
| **Smoking** |  |  |  |  |  |  |  | 0 (non- smoker)  1(current smoker) |  |  |
| **Quality of life (SGRQ)** |  |  |  |  |  |  |  |  | 0 (<=30)  1 (31-49)  2 (50-64)  3 (>=65) |  |
| **Total Score** | 0-3 | 0-3 | 0-10 | 0-15 | 0-14 | 0-12 | 0-9 | 0-8 | 0-9 | 0-6 |
| GOLD=Global initiative for chronic Obstructive Lung Disease; BODE=Body mass index, airflow Obstruction, Dyspnoea and severe Exacerbations; BODE upd.=BODE updated; ADO upd.=Age, Dyspnoea, airflow Obstruction updated; e-BODE=severe acute exacerbation of COPD plus BODE; BODEx=Body mass index, airflow Obstruction, Dyspnoea, severe acute Exacerbation of COPD; DOSE=Dyspnoea, Obstruction, Smoking and Exacerbation frequency; SAFE=Saint George’s Respiratory Questionnaire (SGRQ) score, Air-Flow limitation and Exercise capacity; B-AE-D opt.=Body-mass index, Acute Exacerbations, Dyspnoea, optimised; BMI=body-mass index; FEV1% pred.=forced expiratory volume in 1 s percentage predicted; mMRC=modified Medical Research Council *(*MMRC*)* dyspnea scale; 6MWT=6-minute walk test; CAT=COPD Assessment Test; SGRQ=Saint George’s Respiratory Questionnaire; previous exacerbations are referred to the previous year. | | | | | | | | | | |

# REFERENCES

1. Hosmer DW, Lemeshow S. Goodness of fit tests for the multiple logistic regression model. *Commun. Stat. - Theory Methods* 1980; 9: 1043–1069.

2. Hosmer DW, Hosmer T, Le Cessie S, Lemeshow S. A comparison of goodness-of-fit tests for the logistic regression model. *Stat. Med.* 1997; 16: 965–980.

3. Hosmer DW, Lemeshow S. Applied Logistic Regression. Wiley; 2004.

4. Debray TPA. Meta-analysis of clinical prediction models. 2013.

5. Kerr KF, Wang Z, Janes H, McClelland RL, Psaty BM, Pepe MS. Net reclassification indices for evaluating risk prediction instruments: a critical review. *Epidemiology* 2014; 25: 114–121.

6. Harrell FE. Regression Modelling Strategies. Bickel P, Diggle P, Feinberg SE, Gather U, Olkin I, Zeger S, editors. Springer - Statistics for Biology and Health; ISBN: 978-3-319-19424-0; 2015.

7. Steyerberg EW. Clinical Prediction Models. Gail M, Krickeberg K, Sarnet J, Tsiatis A, Wong W, editors. Springer - Statistics for Biology and Health - ISBN: 978-1-4419-2648-7; 2010.

8. Hanley JA, McNeil BJ. A Method of Comparing the Areas under Receiver Operating Characteristic Curves Derived from the Same Cases. *Radiology* 1983; 148: 839–843.

9. Zou GY, Yue L. Using confidence intervals to compare several correlated areas under the receiver operating characteristic curves. *Stat. Med.* 2013; 32: 5077–5090.

10. Debray TPA, Damen JAAG, Snell KIE, Ensor J, Hooft L, Reitsma JB, Riley RD, Moons KGM. A guide to systematic review and meta-analysis of prediction model performance. *Bmj* 2017; : i6460.

11. Franchini a. J, Dias S, Ades a. E, Jansen JP, Welton NJ. Accounting for correlation in network meta-analysis with multi-arm trials. *Res. Synth. Methods* 2012; 3: 142–160.

12. Pennells L, Kaptoge S, White IR, Thompson SG, Wood AM. Assessing risk prediction models using individual participant data from multiple studies. *AJE (American J. Epidemiol.* 2014; 179: 621–632.

13. Higgins J, Whitehead A. Borrowing strength from external trials in a meta-analysis. *Stat. Med.* 1996; 15: 2733–2749.

14. Pepe MS. The Statistical Evaluation of Medical Tests for Classification and Prediction. Oxford University Press; 2004.

15. Efron B, Tibshirani RJ. An Introduction to the Bootstrap. Chapman & Hall/CRC; 1994.

16. Lu G, Ades AE. Modeling between-trial variance structure in mixed treatment comparisons. *Biostatistics* 2009; 10: 792–805.

17. Dias S, Welton NJ, Marinho VCC, Salanti G, Higgins J, Ades AE. Estimation and adjustment of bias in randomized evidence by using mixed treatment comparison meta-analysis. *J. R. Stat. Soc. Ser. A Stat. Soc.* 2010; 173: 613–629.

18. Sauter R, Held L. Network meta-analysis with integrated nested Laplace approximations. *Biometrical J.* 2015; 57: 1038–1050.

19. Lumley T. Network meta-analysis for indirect treatment comparisons. *Stat. Med.* 2002; 21: 2313–2324.

20. Lu G, Ades AE. Combination of direct and indirect evidence in mixed treatment comparisons. *Stat. Med.* 2004; 23: 3105–3124.

21. Lu G, Ades a. E. Assessing Evidence Inconsistency in Mixed Treatment Comparisons. *J. Am. Stat. Assoc.* 2006; 101: 447–459.

22. Salanti G, Higgins J, Ades a E, Ioannidis JP a. Evaluation of networks of randomized trials. *Stat. Methods Med. Res.* 2008; 17: 279–301.

23. Sutton AJ, Higgins JPT. Recent developments in meta-analysis. *Stat. Med.* 2008; 27: 625–650.

24. Efthimiou O, Debray TP a, van Valkenhoef G, Trelle S, Panayidou K, Moons KGM, Reitsma JB, Shang A, Salanti G. GetReal in network meta-analysis: a review of the methodology. *Res. Synth. Methods* 2016; .

25. Berkey CS, Hoaglin DC, Antczak-Bouckoms A, Mosteller F, Colditz GA. Meta-analysis of multiple outcomes by regression with random effects. *Stat. Med.* 1998; 17: 2537–2550.

26. Lu G, Welton NJ, Higgins J, White IR, Ades AE. Linear inference for mixed treatment comparison meta-analysis: A two-stage approach. *Res. Synth. Methods* 2011; 2: 43–60.

27. Jackson D, Barrett JK, Rice S, White IR, Higgins J. A design-by-treatment interaction model for network meta-analysis with random inconsistency effects. *Stat. Med.* 2014; 33: 3639–3654.

28. Jackson D, Boddington P, White IR. The design-by-treatment interaction model: a unifying framework for modelling loop inconsistency in network meta-analysis. *Res. Synth. Methods* 2016; 7: 329–332.

29. Higgins J, Jackson D, Barrett JK, Lu G, Ades AE, White IR. Consistency and inconsistency in network meta-analysis: concepts and models for multi-arm studies. *Res. Synth. Methods* 2012; 3: 98–110.

30. Kessels AG, Riet G, Puhan MA, Kleijnen J, Bachmann LM, Minder C. A simple regression model for network meta-analysis. *OA Epidemiol.* 2013; : 1–8.

31. Dias S, Welton NJ, Caldwell DM, Ades a. E. Checking consistency in mixed treatment comparison meta-analysis. *Stat. Med.* 2010; 29: 932–944.

32. Bucher HC, Guyatt GH, Griffith LE, Walter SD. The results of direct and indirect treatment comparisons in meta-analysis of randomized controlled trials. *J. Clin. Epidemiol.* 1997; 50: 683–691.

33. Rücker G, Schwarzer G, Krahn U, König J. netmeta: Network Meta-Analysis using Frequentist Methods. 2016.

34. R Core Team. R: A language and environment for statistical computing. Vienna, Austria: R Foundation for Statistical Computing; 2016.

35. StataCorp. Stata Statistical Software: Release 13. College Station, TX: StataCorp LP; 2013.

36. White IR. Multivariate random-effects meta-regression: Updates to mvmeta. *Stata J.* 2011; 11: 255–270.

37. Soriano JB, Lamprecht B, Ramírez AS, Martinez-Camblor P, Kaiser B, Alfageme I, Almagro P, Casanova C, Esteban C, Soler-Cataluña JJ, De-Torres JP, Miravitlles M, Celli BR, Marin JM, Puhan MA, Sobradillo P, Lange P, Sternberg AL, Garcia-Aymerich J, Turner AM, Han MK, Langhammer A, Leivseth L, Bakke P, Johannessen A, Roche N, Sin DD. Mortality prediction in chronic obstructive pulmonary disease comparing the GOLD 2007 and 2011 staging systems: a pooled analysis of individual patient data. *Lancet Respir. Med.* 2015; 3: 443–450.

38. Cipriani A, Barbui C, Salanti G, Rendell J, Brown R, Stockton S, Purgato M, Spineli LM, Goodwin GM, Geddes JR. Comparative efficacy and acceptability of antimanic drugs in acute mania: A multiple-treatments meta-analysis. *Lancet* Elsevier Ltd; 2011; 378: 1306–1315.

39. Puhan MA, Schunemann HJ, Murad MH, Li T, Brignardello-Petersen R, Singh J a., Kessels AG, Guyatt GH. A GRADE Working Group approach for rating the quality of treatment effect estimates from network meta-analysis. *Bmj* 2014; 349: g5630–g5630.

40. Palmer SC, Mavridis D, Nicolucci A, Johnson DW, Tonelli M, Craig JC, Maggo J, Gray V, Berardis G De, Ruospo M, Natale P, Saglimbene V, Badve S V, Cho Y, Burke M, Faruque L, Lloyd A. Comparison of Clinical Outcomes and Adverse Events Associated With Glucose-Lowering Drugs in Patients With Type 2 Diabetes A Meta-analysis. *JAMA - J. Am. Med. Assoc.* 2016; 316: 313–324.

41. Cipriani A, Furukawa TA, Salanti G, Geddes JR, Higgins J, Churchill R, Watanabe N, Nakagawa A, Omori IM, McGuire H, Tansella M, Barbui C. Comparative efficacy and acceptability of 12 new-generation antidepressants: a multiple-treatments meta-analysis. *Lancet* Elsevier Ltd; 2009; 373: 746–758.

42. Wickham H. ggplot2: Elegant Graphics for Data Analysis. New York: Springer-Verlag; 2009.

43. Caldwell DM, Ades a E, Higgins J. Simultaneous comparison of multiple treatments: combining direct and indirect evidence. *Bmj* 2005; 331: 897–900.

44. Borenstein M, Hedges LV., Higgins J, R. RH. Introduction to Meta-Analysis. Wiley; 2011.

45. Cipriani A, Higgins J, Geddes JR, Salanti G. Research and Reporting Methods Annals of Internal Medicine Conceptual and Technical Challenges in Network Meta-analysis. 2013; .

46. Donegan S, Williamson P, Gamble C, Tudur-Smith C. Indirect Comparisons: A Review of Reporting and Methodological Quality. *PLoS One* 2010; 5: e11054.

47. Song F, Loke YK, Walsh T, Glenny A-M, Eastwood AJ, Altman DG. Methodological problems in the use of indirect comparisons for evaluating healthcare interventions: survey of published systematic reviews. *Bmj* 2009; 338: 1–7.

48. Salanti G. Indirect and mixed-treatment comparison, network, or multiple-treatments meta-analysis: many names, many benefits, many concerns for the next generation evidence synthesis tool. *Res. Synth. Methods* 2012; 3: 80–97.

49. Dias S, Welton NJ, Sutton AJ, Caldwell DM, Lu G, Ades a E. Evidence synthesis for decision making 4: inconsistency in networks of evidence based on randomized controlled trials. *Med. Decis. Making* 2013; 33: 641–656.

50. Debray TPA, Vergouwe Y, Koffijberg H, Nieboer D, Steyerberg EW, Moons KGM. A new framework to enhance the interpretation of external validation studies of clinical prediction models. *J. Clin. Epidemiol.* Elsevier Inc; 2015; 68: 279–289.

51. Evaluation T. Academia and Clinic Spectrum Bias or Spectrum Effect ? Subgroup Variation in Diagnostic. *Ann. Intern. Med.* 2002; : 598–603.

52. Ransohoff D, Feinstein A. Problems of spectrum and bias in evaluating the efficacy of diagnostic tests. *N. Engl. J. Med.* 2010; .

53. Riley RD, Ensor J, Snell KIE, Debray TPA, Altman DG, Moons KGM, Collins GS. External validation of clinical prediction models using big datasets from e-health records or IPD meta-analysis: opportunities and challenges. *Bmj* 2016; 353: i3140.

54. Vergouwe Y, Moons KGM, Steyerberg EW. External validity of risk models: Use of benchmark values to disentangle a case-mix effect from incorrect coefficients. *AJE (American J. Epidemiol.* 2010; 172: 971–980.

55. Cox DR. Two Further Applications of a Model for Binary Regression. *Biometrika* 1958; 45: 562–565.

56. Hanley JA, McNeil BJ. The Meaning and Use of the Area under a Receiver Operating ( ROC ) Curvel Characteristic. *Radiology* 1982; 143: 29–36.

57. Thompson SG, Higgins J. How should meta-regression analyses be undertaken and interpreted? *Stat. Med.* 2002; 21: 1559–1573.

58. Rubin DB. Multiple Imputation for Nonresponse in Surveys. Harvard Univ. Wiley Serie in Probability and Mathematical Statistics; 1987.

59. Harrell FE. Regression Modeling Strategies. Springer; 2015.

60. Van Buuren S, Groothuis-Oudshoorn K. Multivariate Imputation by Chained Equations. *J. Stat. Softw.* 2011; 45: 1–67.

61. Jolani S, Debray TPA, Koffijberg H, van Buuren S, Moons KGM. Imputation of systematically missing predictors in an individual participant data meta-analysis: A generalized approach using MICE. *Stat. Med.* 2015; 34: 1841–1863.

62. Decramer M, Vogelmeier C, Agustí AG, Bourbeau J, Celli BR, Chen R, Criner GJ, Frith P, Halpin D, Varela MVL, Nishimura M. Global Strategy for the Diagnosis, Management, and Prevention of Chronic Obrstructive Pulmonary Disease (updated 2017). *Glob. Initiat. Chronic Obstr. Lung Dis.* 2011; .

63. Celli BR, Cote CG, Marin JM, Casanova C, Montes de Oca M, Mendez R a, Pinto Plata V, Cabral HJ. The body-mass index, airflow obstruction, dyspnea, and exercise capacity index in chronic obstructive pulmonary disease. *N. Engl. J. Med.* 2004; 350: 1005–1012.

64. Puhan MA, Garcia-Aymerich J, Frey M, ter Riet G, Antó JM, Agusti A, Gómez FP, Rodríguez-Roisín R, Moons KGM, Kessels AG, Held U. Expansion of the prognostic assessment of patients with chronic obstructive pulmonary disease: the updated BODE index and the ADO index_Puhan_2009_210. *Lancet* Elsevier Ltd; 2009; 374: 704–711.

65. Puhan MA, Hansel NN, Sobradillo P, Enright P, Lange P, Hickson D, Menezes AM, Riet G Ter, Held U, Domingo-Salvany A, Mosenifar Z, Antó JM, Moons KGM, Kessels AG, Garcia-Aymerich J. Large-scale international validation of the ADO index in subjects with COPD: an individual subject data analysis of 10 cohorts_PUHAN_BMJ_2012_9. *Bmj* 2012; 2: e002152.

66. Soler-Cataluña JJ, Martinez-Garcia MA, Sanchez LS, Tordera MP, Sanchez PR. Severe exacerbations and BODE index: two independent risk factors for death in male COPD patients. *Respir. Med.* Hospital General de Requena, Unidad de Neumologia, Servicio de Medicina Interna, Paraje Casablanca s/n., 46340 Requena, Valencia, Spain. jjsoler@telefonica.net; 2009; 103: 692–699.

67. Jones RC, Donaldson GC, Chavannes NH, Kida K, Dickson-Spillmann M, Harding S, Wedzicha JA, Price D, Hyland ME. Derivation and validation of a composite index of severity in chronic obstructive pulmonary disease: the DOSE Index. *Am. J. Respir. Crit. Care Med.* 2009; 180: 1189–1195.

68. Azarisman MS, Fauzi MA, Faizal MP, Azami Z, Roslina AM, Roslan H. The SAFE (SGRQ score, air-flow limitation and exercise tolerance) Index: a new composite score for the stratification of severity in chronic obstructive pulmonary disease. *Postgrad. Med. J.* Department of Medicine, International Islamic University Malaysia, Jalan Hospital Campus, Kuantan, Pahang, Malaysia. risman1973@hotmail.com; 2007; 83: 492–497.

69. Boeck L, Soriano JB, Brusse-Keizer M, Blasi F, Kostikas K, Boersma W, Milenkovic B, Louis R, Lacoma A, Djamin R, Aerts J, Torres A, Rohde G, Welte T, Martinez-Camblor P, Rakic J, Scherr A, Koller M, Van Der Palen J, Marin JM, Alfageme I, Almagro P, Casanova C, Esteban C, Soler-Cataluña JJ, De-Torres JP, Miravitlles M, Celli BR, Tamm M, Stolz D. Prognostic assessment in COPD without lung function: The B-AE-D indices. *Eur. Respir. J.* 2016; 47: 1635–1644.
